# Efficacy and safety of praziquantel and dihydroartemisinin piperaquine combination for treatment and control of intestinal schistosomiasis: A randomized, non-inferiority clinical trial

**DOI:** 10.1371/journal.pntd.0008619

**Published:** 2020-09-23

**Authors:** Rajabu Hussein Mnkugwe, Omary Minzi, Safari Kinung’hi, Appolinary Kamuhabwa, Eleni Aklillu

**Affiliations:** 1 Department of Clinical Pharmacology, School of Medicine, Muhimbili University of Health and Allied Sciences, Dar es Salaam, Tanzania; 2 Division of Clinical Pharmacology, Department of Laboratory Medicine, Karolinska University Hospital-Huddinge, Karolinska Institutet, Stockholm, Sweden; 3 Department of Clinical Pharmacy and Pharmacology, School of Pharmacy, Muhimbili University of Health and Allied Sciences, Dar es Salaam, Tanzania; 4 National Institute for Medical Research (NIMR), Mwanza Research Centre, Mwanza, Tanzania; Imperial College London, Faculty of Medicine, School of Public Health, UNITED KINGDOM

## Abstract

**Background:**

Despite the reported success in reducing morbidity, praziquantel alone is insufficient for the control and elimination of schistosomiasis, partly due to its poor efficacy against the juvenile worms. Artemisinin derivatives are effective against juvenile worms but are less effective against adult worms. We compared the safety and efficacy of praziquantel and Dihydroartemisinin-piperaquine combination against the standard praziquantel alone for treatment of intestinal schistosomiasis.

**Methods:**

In this randomized, open-label, non-inferiority trial, 639 *Schistosoma mansoni* infected children were enrolled and randomized to receive either praziquantel alone or praziquantel plus Dihydroartemisinin-piperaquine combination. Two stool samples were collected on consecutive days at baseline, 3 and 8 weeks post-treatment and analyzed using thick smear Kato Katz method. Efficacy was assessed by cure and egg reduction rates at 3 and 8 weeks post-treatment. Adverse events were assessed within four hours of drugs intake. The primary outcome was cure rates at 8 weeks of post-treatment. Secondary outcomes were egg reduction rates at 8 weeks of post-treatment and treatment-associated adverse events.

**Results:**

At 3 weeks of post-treatment, cure rates were 88.3% (263/298, 95% CI = 84.1%– 91.4%) and 81.2% (277/341, 95% CI = 76.7%– 85.0%) for the combination therapy and praziquantel alone, respectively (*p* < 0.01, odds ratio (OR) = 1.74, 95% CI of OR = 1.11 to 2.69). At 8 weeks, there was a significant drop in the cure rates in praziquantel alone group to 63.9% (218/341, 95% CI = 58.7%– 68.8%) compared to 81.9% (244/298, 95% CI = 77.1%– 85.8%) in the combination therapy group (*p* < 0.0001, OR = 2.55, 95%CI of OR = 1.75 to 3.69). Egg reduction rates at 8 weeks post-treatment were significantly higher in the combination therapy group 93.6% (95% CI = 90.8%– 96.4%) compared to 87.9% (95% CI = 84.4%– 91.4%) in the praziquantel only group (*p* = 0.01). On both Univariate and Multivariate regression analysis, type of treatment received was a significant predictor of cure at week 8 post-treatment. Overall, 30.8% (95% CI = 27.2%– 34.4%) of the study participants experienced mild and transient treatment-associated adverse events, post-treatment abdominal pain (27.1%) being the most common adverse event observed. There was no significant difference in the overall occurrence of adverse events between the two treatment groups.

**Conclusion:**

Praziquantel and Dihydroartemisinin piperaquine combination therapy is safe, and more efficacious compared to praziquantel alone for the treatment of intestinal schistosomiasis. Further studies are needed to explore if the combination therapy can be considered as an option for mass drug administration to control and eventually eliminate schistosomiasis.

## Author summary

Schistosomiasis is a Neglected Tropical Disease, which is caused by a parasite called Schistosoma. The disease is mainly manifested by bloody urine (urinary schistosomiasis) and bloody diarrhea (intestinal schistosomiasis). Schistosomiasis is usually asymptomatic during early infection but, if not treated, may cause devastating long-term complications, including poor growth and cognitive development in children, portal hypertension, and cancers. For several years, children in Sub Saharan Africa have been receiving targeted mass praziquantel treatments for the control and elimination of schistosomiasis. Despite the reported success in reducing morbidity, single dose praziquantel alone treatment is apparently insufficient for elimination of schistosomiasis in endemic countries. This is partly due to its poor activity against the immature/juvenile stage of the parasite. Previous studies reported that artemisinin derivatives are effective against juvenile worms. In this study, we explored if combination therapy of praziquantel and Dihydroartemisinin-piperaquine would increase the efficacy by targeting to kill both developmental stages (matured and juvenile worms) of the parasite. In a randomized clinical trial, we compared the efficacy and safety of combination therapy of praziquantel and Dihydroartemisinin-piperaquine against the standard praziquantel alone for the treatment of intestinal schistosomiasis in a rural endemic setting in North-Western Tanzania. Our findings indicate that praziquantel and Dihydroartemisinin piperaquine combination therapy is safe and more effective than praziquantel alone for the treatment of intestinal schistosomiasis.

## Introduction

Schistosomiasis is among the Neglected Tropical Diseases (NTDs) that continue to be of major public health importance, especially among school-going children in Sub Saharan Africa (SSA) [1]. This region harbors more than 90% of the 250 million cases reported globally [2, 3], and more than 280,000 deaths annually [2, 4]. Among parasitic diseases, schistosomiasis is second only to malaria in terms of morbidity and mortality, particularly among children [5]. It is estimated that schistosomiasis causes up to 3.31 million disability-adjusted life years (DALYs) [2]. Despite the ongoing targeted mass praziquantel administration as intervention, the disease is still prevalent in Tanzania, especially among school children, and endemic throughout the country, making Tanzania the second country next to Nigeria in terms of disease burden among African countries [5]. Both *Schistosoma haematobium* (Urogenital schistosomiasis) and *Schistosoma mansoni* (Intestinal schistosomiasis) are endemic in Tanzania, with the latter being highly prevalent around the Lake Victoria Zone [6, 7].

To date, praziquantel (PZQ) is the only available and recommended drug by the World Health Organization (WHO) for the treatment and control of schistosomiasis worldwide [8]. It is efficacious against all Schistosoma species, including *Schistosoma haematobium* and *Schistosoma mansoni* [8]. Given as a single dose (40mg/kg body weight) in mass drug administration (MDA) programs, PZQ has been successful in reducing morbidity and mortality, especially among the risk groups such as school-going children in endemic settings [9]. In 2012, the WHO recommended to its member states to think beyond the control of disease morbidity to elimination [10, 11]. Despite the reported success in reducing morbidity [12], single dose PZQ alone seems to be not an optimal treatment to eliminate schistosomiasis in endemic settings [6, 13]. The reason for this shortcoming, among others, is its poor activity against the immature/juvenile stage of the parasite [14]. In the juvenile stage of the parasite, PZQ target might not have developed since PZQ is postulated to act mainly by destroying the outer tegument of the parasite [15]. Therefore, MDA with PZQ leaves behind the immature parasites that grow to mature parasites and start laying eggs, and hence the prevalence and intensity of infection go back to the pre-treatment levels to continue transmission. Failure to control the disease causes a negative impact on the human development index (HDI) and delays in the achievement of the sustainable development goals (SDGs) such as reduction of poverty (SDG1), ensure healthy lives and promote well-being for all at all ages (SDG3) and attaining quality education to children (SDG4) [16].

Vaccines are the best option for schistosomiasis prevention, but potential candidates are still on clinical trials [14, 17]. Therefore, repurposing or optimizing the use of available drugs remains the option in the era of schistosomiasis control and elimination worldwide. Few randomized clinical trials (RCTs) have been conducted for prevention and control of schistosomiasis [18], and several strategies have been tested in an attempt to optimize schistosomiasis treatment outcomes including use of higher PZQ doses [19, 20], repeated PZQ doses [21–24], and combination of PZQ with other antischistosomal drugs such as oxamniquine [25]. However, each strategy has shown its weaknesses or limitations such as safety concern, cost-effectiveness, or risk of parasite resistance, and thus necessitating the need for exploring other alternatives strategies.

Combination drug therapy, like in other infectious diseases such as HIV, tuberculosis, and malaria, remains a key strategy in optimizing schistosomiasis treatment outcomes and also reduce the risk of development of resistance against PZQ [26]. A combination of PZQ and oxamniquine has been tested but the findings have not shown convincing evidence of superior benefit to PZQ alone [25, 27]. Not only is oxamniquine ineffective against the immature stage of the parasite, but also it does not act on all Schistosoma species (*S*. *mansoni* only) [25]. A feasible strategy is to combine PZQ with a drug that has efficacy against the immature stages of the parasites. Artemisinin and its derivatives (Artemether, Artesunate and Dihydroartemisinin) have demonstrated that property apart from their antimalarial activity [14]. Artemisinin in combination with PZQ have been tested in treating schistosomiasis and shown success by improving the cure rates and thereby reducing disease morbidity [28–30]. A concern is raised on its use as monotherapy in settings where malaria is also endemic due to the risk of developing artemisinin resistance in malaria parasites. However, artemisinin-based combination therapy (ACTs) such as Artemether Lumefantrine (ALU) or Dihydroartemisinin piperaquine (DHP) provides a better option for use in combination with PZQ since the risk of artemisinin malaria parasite resistance is greatly reduced as artemisinin is protected by the long-acting partner drug (Lumefantrine or Piperaquine) [31]. In fact, the use of ACTs for malaria treatment in patients co-infected with schistosomiasis produced 100% schistosomiasis cure rates, but the small number of study participants being the only limitation in those studies [32–34]. DHP is a first-line antimalarial drug recommended by the WHO for the management of uncomplicated malaria worldwide [35]. To our knowledge, PZQ and DHP combination has not yet been tested for the treatment of intestinal schistosomiasis. The objective of this work was to investigate whether praziquantel and Dihydroartemisinin-piperaquine combination therapy increases efficacy by targeting both matured and juvenile worms compared to the standard praziquantel alone for the treatment of intestinal schistosomiasis. We report the first randomized clinical trial involving the use of combination therapy of praziquantel and artemisinin-based combination therapy such as Dihydroartemisinin piperaquine for treatment of intestinal schistosomiasis in rural north-western Tanzania.

## Methods

### Study area

This study was conducted in Busega district, Simiyu region, North-Western Tanzania, between February 2017 and January 2018 in collaboration with the National Institute for Medical Research (NIMR), Mwanza Research Centre. According to the 2012 population and housing census data, the district had a population of about 203,597 people [36]. School children residing in Nyamikoma village were included in this study. The village is a rural endemic setting for intestinal schistosomiasis located along the shores of Lake Victoria. This study site was purposely selected for the trial because of the high disease endemicity and accessibility to NIMR Mwanza Research Centre laboratory.

### Study design and population

This was a randomized, open-label, non-inferiority clinical trial aimed at assessing the efficacy and safety of a combination of PZQ and DHP versus the standard PZQ alone for the treatment and control of intestinal schistosomiasis. The study enrolled school-going children (aged 7–17 years) residing in Nyamikoma village and attending Fogofogo primary school in Busega district, north-western Tanzania.

### Sample size calculation

The required sample size for this study was calculated using the formula for comparing two proportions [37]. For PZQ alone group (control), we used the cure rate of 68% at week 8 of post-PZQ alone treatment reported by a recent study that compared single versus double doses of PZQ for the treatment of intestinal schistosomiasis [23]. Assumptions were; a combination of PZQ and DHP (test group) will produce cure rates of 80%, the significance level was set at 95%, and power of 90% and a 10% loss to follow-up was considered. The minimum estimated sample size was 305 infected children in each treatment group.

#### Inclusion and exclusion criteria

School children who reside in the selected study village and attending Fogofogo primary school, whose parents/guardians consented for their children to participate, and children who provided assent to participate were enrolled. Children who had received PZQ tablets within the past 6 months and/or antimalarial drug(s) e.g. ALU (Coartem and Artefan) or DHP products (Artequick, D artepp, Duo cotecxin or Ridmal) within the past 2 weeks before commencement of data collection were excluded.

### Data collection procedures

#### Sociodemographic characteristics

Sociodemographic and other clinical information of the study participants were collected using individual case record forms (CRFs). Information collected includes age (from school registry), sex and grade of the study participants.

#### Stool samples collection, processing and examination

Fresh stool samples were collected from study participants during both screening (diagnosis) and follow-up visits (at 3 weeks and 8 weeks post-treatment). To increase the sensitivity of the thick smear Kato Katz method [38], two fresh stool samples were collected on two consecutive days during each study visit. On each day, children were given a stool collection container and properly trained on how to collect stool samples. From each stool sample, two Kato Katz thick smears were prepared using a 41.7mg template in a temporary field laboratory by well-experienced technicians from NIMR Mwanza Research Centre [39]. The prepared Kato Katz smears were transported to NIMR Mwanza laboratory and were examined under a light microscope on the following day by two well-trained and experienced technicians. The examination of the smears was done on the following day in order to increase the staining of the eggs for proper visibility. The slides were double read by the two technicians independently. Egg count for each participant was obtained by taking the average number of eggs recorded from each Kato Katz slide. The average egg count were multiplied by 24 to get eggs count per gram of stool (epg) for each participant, which is the measure of infection intensity [39]. The infection intensity were classified according to the WHO criteria; Light infection (1–99 epg), moderate infection (100–399 epg) and heavy infection (≥ 400 epg) [9]. Microscopic examination for other helminths (e.g. soil-transmitted helminths), which is time-dependent (within 1 hour of smear preparation) was not in the scope of this study. Another independent technician re-examined 10% of the slides from both positive and negative results for quality assurance [40, 41].

#### Haemoglobin concentration and anthropometric measurements

Haemoglobin concentration (Hb conc) in g/dL was determined from a finger prick venous blood using a HemoCue Hb 201+ machine (HemoCue AB Angelholm, Sweden) [42]. Anaemia was defined as Hb conc < 11.5g/dL and was classified by severity using the WHO guideline, whereas a Hb conc < 8g/dL was considered as severe anaemia [43].

Bodyweight and height for each study participant were measured in kilograms (kgs) and centimeter (cm), respectively. A calibrated digital weighing scale and portable stadiometer were used to record the readings to the nearest 0.1 kg and 0.1 cm for body weight and height, respectively. The collected anthropometric data were converted to height for age Z score (HAZ) and body mass index (BMI) for age Z score (BAZ) using the WHO Anthro-Plus software version 1.0.4 [44]. Children whose HAZ and BAZ scores were below 2 standard deviations (SD) were considered stunted and wasted, respectively.

#### Randomization, treatment and safety monitoring

PZQ tablets were received as a donation from the Tanzanian NTDs Control Program. DHP tablets (D-Artepp) were received as a donation from Guilin Pharmaceutical Co. Ltd, China (registered in Tanzania as TZ14H0310). Children who tested positive for *S*. *mansoni* infection and consented to participate were enrolled in the study. After baseline data collection, study participants were randomized into control or intervention group at a ratio 1:1 using Computer-generated random numbers. The control group received the standard single-dose PZQ alone 40mg/kg bodyweight (Batch BZ6043, S Kant Health Care Ltd, India). The intervention group received single-dose PZQ 40mg/kg body weight plus DHP tablets (40mg Dihydroartemisinin / 320mg piperaquine, batch number S0160103, Guilin Pharmaceutical Co. Ltd, Guangxi, China). The dose of DHP was calculated based on the child’s body weight (in kg) and was given once per day for three consecutive days following the WHO treatment guideline for uncomplicated malaria [35]. Before drug administration, children were given a standardized meal (porridge + biscuits) to reduce the nauseating effect of study drugs as per the WHO recommendation [45]. All drugs were given as directly-observed treatment (DOT). Assessment of treatment-associated adverse events was done within 4 hours of drug administration through close monitoring of the study participants. If present or reported, the adverse events were recorded by the study nurse and reviewed by the study team leader to assess for their severity.

#### Follow-up post-treatment and study outcomes

After treatment, the study participants were followed up for a maximum period of 8 weeks (2 months), during which two follow-up visits were made. The first follow-up was done at 3 weeks (21 days), while the second follow-up was at 8 weeks post-treatment. The 8 weeks visit was chosen because it is around this time (mainly 4–6 weeks) when immature worms get matured and start laying eggs [1, 31].

The primary outcome of the study was efficacy defined by parasitological cure rate for *S*. *mansoni* infection at 8 weeks post-treatment. Secondary outcomes were egg reduction rate and any treatment-associated adverse events observed within 4 hours of drugs intake.

### Data management and statistical analysis

Data were double entered into a database Census and Survey Processing System (CSPro) (US Census Bureau, USA) developed by the Data Management Unit of NIMR Mwanza Research Centre. Entered data were exported to Excel format for cleaning and analyzed using the Statistical Package for Social Sciences (SPSS) for Windows version 20. Descriptive statistics of sociodemographic and baseline characteristics of the study participants were summarized using frequency tables stratified by treatment groups. A comparison of baseline mean egg intensity (epg) between infection intensities (light to heavy infections) and treatment groups was done using two-way ANOVA. Parasitological cure rate (CR) was defined as a proportion of children who were *S*. *mansoni* positive at baseline but turned negative at the post-treatment follow-up visits. Egg reduction rate (ERR) was defined as the proportional reduction in the mean eggs excreted from baseline to post-treatment [46] and calculated according to the recommended formula by the WHO as 100 times [1- (Arithmetic mean epg after treatment/Arithmetic mean epg before treatment)] [45]. Descriptive statistics was also used to analyze the observed treatment-associated adverse events between the treatment groups and summarized in percentages. Contingency table in GraphPad Prism software package version 8.0 was used to calculate the 95% confidence intervals (CI) of CR in each treatment group, and to compare CRs between treatment groups using Chi-square or Fishers Exact test with its odds ratio (OR) and 95% CI of ORs. The modified "N-1" Chi-squared test was used to compare ERR (percentage reduction in eggs count from baseline at 3 and 8 weeks of post-treatment) between treatment groups using the online available MediCal statistical software (https://www.medcalc.org/calc/comparison_of_proportions.php). Univariate and multivariate logistic regression analysis were used to assess for predictors of cure at week 8 post-treatment. Predictor variables with a p-value ≤ 0.2 from the Univariate analysis were included in the multivariate analysis model. A p-value of < 0.05 was considered statistically significant.

### Ethical statement

The trial was registered on the Pan African Clinical Trial Registry with registration number PACTR201612001914353. Ethics approval was granted by the Institutional Review Board of Muhimbili University of Health and Allied Sciences (Ref:2016-5-25/AEC/Vol.X/03) and the Medical Research Coordination Committee of the National Institute of Medical Research, Tanzania (NIMR/HQ/R.8a/Vol.IX/2343). Permission to conduct the study was also granted from relevant Busega district authorities; District Executive Director (DED), District Medical Officer (DMO), District Education Officer (DEO), Village Leaders, and School administration. Finally, written consent from parents/guardians and assent from participating children was obtained.

## Results

### Sociodemographic and baseline characteristics

**Fig 1:**
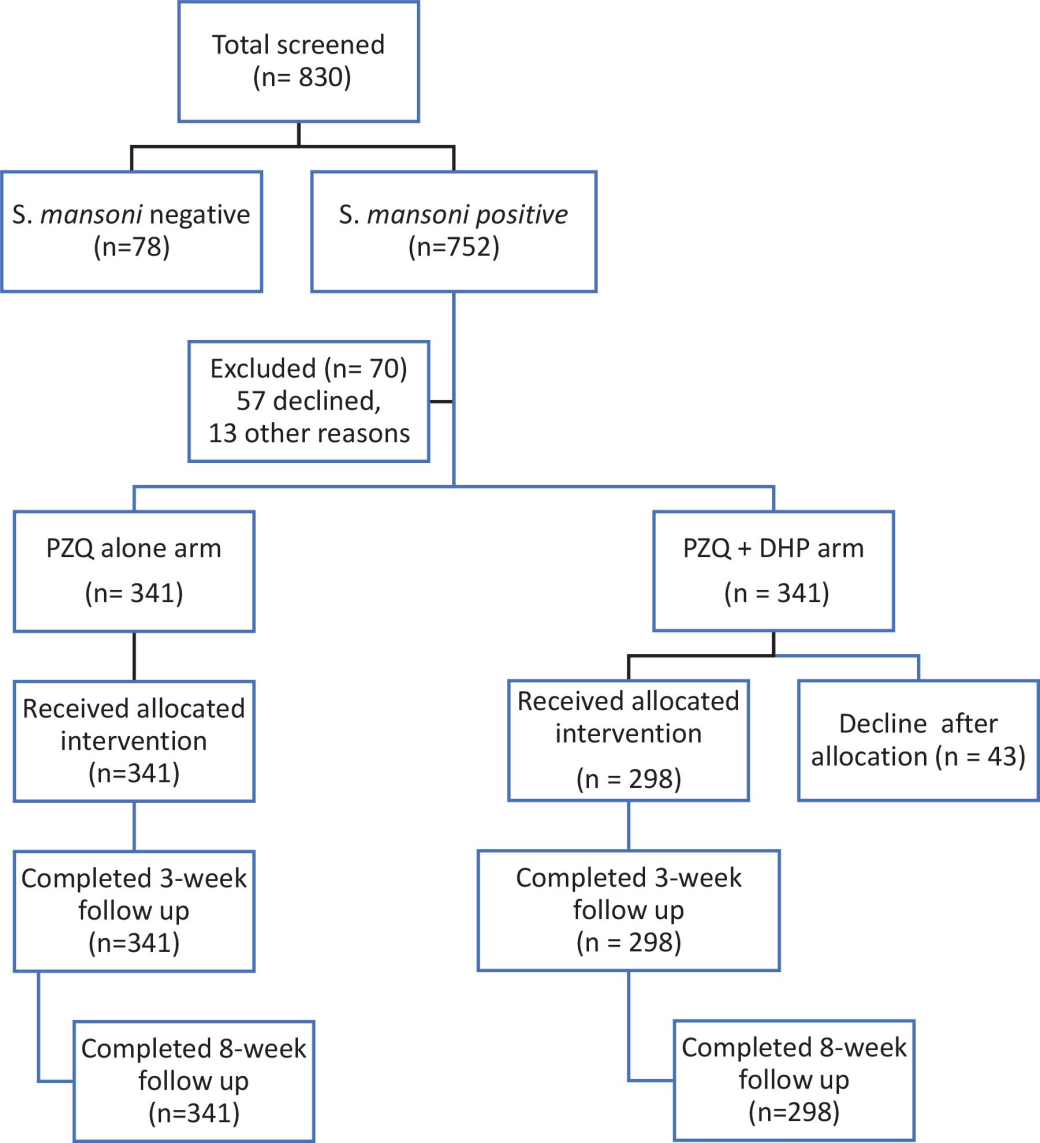
Study flow chart showing screening, treatment allocation, and study follow-up.

At baseline, a total of 830 school children were screened for intestinal schistosomiasis. Of the 830 screened children, 639 (77.0%) children (aged 7–17 years) tested positive for *S*. *mansoni* and consented to participate in the study were enrolled (Fig 1). Overall, females constituted 52.3% (334/639) of all enrolled children. The overall infection intensities among study participants were; Light infection 170/639 (26.6%), moderate infection 280/639 (43.8%) and heavy infection 189/639 (29.6%). After randomization, 341 infected children received PZQ alone and 298 received PZQ + DHP combination therapy. The Sociodemographic and baseline characteristics between the two treatment groups were similar at baseline except for stunting (*p* < 0.001) where stunting was observed more in participants receiving PZQ alone (34.3%) compared to 21.8% in the combination therapy. Table 1 summarizes the sociodemographic and baseline characteristics of the study population by treatment groups.

**Table 1:** Sociodemographic and baseline characteristics of the studied population between treatment groups.

Characteristic	PZQ alone (n = 341) N (%)	PZQ+DHP (n = 298) N (%)	*p*-value
**Age group**			
Mean age +SD	11.7 ± 1.7	11.3 ± 1.9	
≤12 years	235 (68.9)	225 (75.5)	0.06^a^
>12 years	106 (31.1)	73 (24.5)	
**Sex**			
Male	160 (46.9)	145 (48.7)	0.66^a^
Female	181 (53.1)	153 (51.3)	
**Infection intensity**			
Light	87 (25.5)	83 (27.9)	0.80^a^
Moderate	152 (44.6)	128 (43.0)	
Heavy	102 (29.9)	87 (29.2)	
**Haemoglobin concentration**			
Mean Hb concentration ± SD	12.5 ± 1.8	12.3 ± 1.5	0.27^b^
**Stunting (HAZ score)**			
Present	117 (34.3)	65 (21.8)	< 0.001^a^
absent	224 (65.4)	233 (78.2)	
**Wasting (BAZ score)**			
Present	34 (10.0)	31 (10.4)	0.86^a^
Absent	307 (90.0)	267 (89.6)	
**Egg counts/gram of stool (epg)**			
Mean epg + SD	365.1 ± 437.4	376.0 ± 449.6	0.91^c^
Light infection	44.3+26.6	50.4±27.0	0.54^d^
Moderate infection	222.5±81.1	224.0±84.3	
Heavy infection	851.1±525.3	909.7±511.9	

BAZ- body mass index (BMI) for age Z score, HAZ -height for age Z score

a–Chi-square test

b-Student t test

c–Mann Whitney U test

d-Two way ANOVA

### Parasitological cure rates between treatment groups

Overall, at both post-treatment follow-ups, the combination therapy produced significantly higher parasitological cure rates compared to PZQ alone for treatment of intestinal schistosomiasis. At 3 weeks post-treatment, parasitological cure rates were 88.3% (95% CI = 84.1%– 91.4%) and 81.2% (95% CI = 76.7%– 85.0%) in the PZQ + DHP group and PZQ alone, respectively (*χ*^*2*^ = 5.991 *p* = 0.01, odds ratio (OR) = 1.74, 95% CI of OR = 1.11 to 2.69). The difference between treatment groups was 7.1% (95%CI of difference = 1.48%-12.6%). While at 8 weeks, cure rates were 81.9% (95% CI = 77.1%– 85.8%) and 63.9% (95% CI = 58.7%– 68.8%) in the PZQ + DHP and PZQ alone group, respectively (*χ*^*2*^ = 25.584 *p* < 0.001, OR = 2.55, 95%CI of OR = 1.75 to 3.69). The difference between treatment groups was 18% (95%CI of difference = 11.15%-24.55%) (Table 2). There were significant differences in cure rates between 3 weeks post-treatment and 8 weeks post-treatment (*χ*^*2*^ = 241.25 *p* **<** 0.001).

**Table 2:** Comparison of cure rates between treatment groups at 3 weeks and 8 weeks post-treatment.

Post-treatment follow-up	Treatment group	Cured N (%)	Not cured N (%)	*χ*^*2*^ value	Odds ratio (95% CI of OR)	*p*-value
**3 weeks**	PZQ alone	277 (81.2)	64 (18.8)	5.991	1.74 (1.11–2.69)	0.01
	PZQ+DHP	263 (88.3)	35 (11.7)			
**8 weeks**	PZQ alone	218 (63.9)	123 (36.1)	25.584	2.55 (1.75–3.69)	< 0.0001
	PZQ+DHP	244 (81.9)	54 (18.1)			

Within each treatment group, there was no statistically significant association of cure rates with age groups, sex or wasting at both follow-up visits (*p* > 0.05). Likewise, despite the observed differences in stunting at baseline between treatment groups, no significant association was found between cure rates and stunting at both follow-up study visits (*p* > 0.05) (S1 and S2 Tables).

At 3 weeks post-treatment, the combination therapy of PZQ plus DHP produced significantly higher cure rates particularly in light infections (94%) compared to PZQ alone (82.8%) (*χ*^*2*^ = 5.12 *p* = 0.02) whereas the cure rates in moderate to heavy infections were higher in the combination therapy than in PZQ alone but were not significant (*p >*0.05). While at 8 weeks post-treatment, the combination therapy of PZQ and DHP produced significantly higher cure rates at all levels of infection intensities (light to heavy infections) compared to PZQ alone (Table 3).

**Table 3:** Comparison of the cure rates between infection intensities by treatment groups.

Post-treatment follow-up	Infection intensity	PZQ group		PZQ+DHP group		*χ*^*2*^ *value*	*p*-value
		Treated N	Cured %	Treated N	Cured %		
**3 weeks**	Light	87	82.8	83	94.0	5.12	0.02
	Moderate	152	82.9	128	89.8	2.75	0.09
	Heavy	102	77.5	87	80.5	0.252	0.62
**8 weeks**	Light	87	63.2	83	88.0	13.97	< 0.001
	Moderate	152	67.1	128	82.0	7.97	0.01
	Heavy	102	59.8	87	75.9	5.49	0.02

### Predictors of cure at week 8 post-treatment

On both Univariate and multivariate regression analysis, the type of treatment received by study participants was a significant predictor of cure at 8 week post-treatment (Table 4).

**Table 4:** Univariate and multivariate regression analysis of the predictors of cure at week 8 post-treatment.

Variable	Categories	Cured N (%)	Univariate analysis			Multivariate analysis		
			OR	95% CI	*p*- value	aOR	95% CI	*p*- value
Treatment group	PZQ+DHP	244 (81.9)	0.39	0.27–0.58	**<** 0.001	0.38	0.26–0.55	**<** 0.001
	PZQ	218 (63.9)	1^a^			1^a^		
Age (years)	≤ 12	326 (70.9)	1.30	0.87–1.94	0.19	1.45	0.96–2.19	0.08
	>12	136 (76.0)	1^a^			1^a^		
Sex	Male	226 (74.1)	0.84	0.59–1.19	0.33			
	Female	236 (70.7)	1^a^					
Anaemia	Anaemic	118 (74.7)	0.85	0.57	0.44			
	Not anaemic	344 (71.5)	1^a^					
Stunting (HAZ)	Present	134 (73.6)	0.91	0.62–1.34	0.64			
	Absent	328 (71.8)	1^a^					
Wasting (BAZ)	Present	53 (81.5)	0.56	0.29–1.08	0.08	0.59	0.30–1.16	0.13
	Absent	409 (71.3)	1^a^			1^a^		
Baseline infection intensity	Light	128 (75.3)	0.67	0.42–1.07	0.09	0.67	0.42–1.08	0.10
	Moderate	207 (73.9)	0.72	0.48–1.08	0.12	0.69	0.46–1.06	0.09
	Heavy	127 (67.2)	1^a^			1^a^		

N—Total number of participants belong to the category, 1^a:^ reference category, aOR—adjusted odds ratio

### Egg reduction rates between treatment groups

At both follow up visits, the arithmetic and geometric means of egg counts of were lower in the combination therapy compared to PZQ alone. Geometric means of egg counts are presented in supplement file (S3 Table). At 3 weeks follow-up visit, PZQ + DHP combination produced almost similar ERR (95.3%, 95% CI = 92.9%– 97.7%) to that of PZQ alone (95.0%, 95% CI = 92.7%– 97.3%) (*p* = 0.86, 95% CI for the difference in ERR = -3.254% to 3.719%). However, at 8 weeks follow-up, ERR were significantly higher in the PZQ + DHP combination therapy group (93.6%, 95% CI = 90.8%– 96.4%) compared to PZQ alone group (87.9%, 95% CI = 84.4%– 91.4%) (*p* = 0.01, 95% CI for the difference in ERR = 1.14% to 10.17%). Table 5 presents the arithmetic means of eggs counts at baseline, and follow-up visits among those who were not cured, and their respective ERR stratified by treatment group.

**Table 5:** Comparison of the egg reduction rates between treatment groups at 3 weeks and 8 weeks post-treatment.

	Visit	PZQ alone	PZQ + DHP	*p*-value
Eggs count /gram of stool (Arithmetic mean ± SD)	At baseline	365.1±437.4	375.9±449.5	
	At 3 weeks visit	18.2±65.6	17.6±121.2	
	At 8 weeks visit	44.0±116.5	24.0±72.9	
Egg Reduction Rate (%)	at 3 weeks visit	95.0	95.3	0.86
	at 8 weeks visit	87.9	93.6	0.01

### Treatment-associated adverse events

Of the 639 treated children, 197 (30.8%, 95% CI = 27.2%– 34.4%) experienced adverse events within 4 hours of drug administration. In the PZQ alone group, 97/341 (28.4%, 95% CI = 23.7%– 33.3%) of the children experienced adverse events while in the PZQ + DHP group 100/298 (33.6%, 95% CI = 28.2%– 38.9%) of the children experienced adverse events. Overall there was no significant difference in the occurrence of adverse events between the two treatment groups (*χ*^*2*^ = 2.013, *p* **=** 0.16). Abdominal pain 173/639 (27.1%) and vomiting (2 hours post-drug administration) 22/639 (3.4%) were the adverse events observed among treated children. Vomiting was observed more in patients who received combination therapy (5.4%) than PZQ alone (1.8%). Almost all adverse events were mild and transient. Table 6 summarizes the frequency of observed adverse events and comparison between treatment groups.

**Table 6:** Comparison of the observed treatment-related adverse events between treatment groups.

Adverse events	Overall n = 639	PZQ alone (n = 341)	PZQ+DHP (n = 298)	*p*-value
	Yes N (%)	Yes N (%)	Yes N (%)	
Total adverse events	197 (30.8)	97 (28.4)	100 (33.6)	0.16
Abdominal pain	173 (27.1)	91 (26.7)	82 (27.5)	0.82
Vomiting	22 (3.4)	6 (1.8)	16 (5.4)	0.01
Nausea	2 (0.3)	0 (0.0)	2 (0.7)	0.12

## Discussion

In this randomized, open-label, non-inferiority clinical trial, we evaluated the efficacy and safety of combination therapy of PZQ and DHP against the standard PZQ alone for the treatment of intestinal schistosomiasis in a rural endemic setting in North-Western Tanzania. PZQ is efficacious against adults matured parasites but is less effective against juvenile worms [14]. We hypothesized that combining ACTs with PZQ would complement and potentially add to the killing effect of both immature and matured stages of the parasite to prevent transmission as suggested previously [31, 47]. Several ACTs exist, but DHP was chosen in this study due to its multifactorial advantages including: (i) DHP has not been extensively used for malaria treatment in Tanzania compared to other first-line antimalarial drugs such as ALU, (ii) cost-effectiveness and easy schedule of DHP administration (once a day) compared to ALU and (iii) its long duration of post-treatment prophylaxis which will offer protection to children against malaria who remain to be a vulnerable population in SSA. To our knowledge this is the first clinical trial to investigate the efficacy and safety of PZQ and ACTs combination against the standard PZQ alone for the treatment of intestinal schistosomiasis. Our results indicate that the combination therapy of PZQ and DHP is safe and more efficacious than PZQ alone for the treatment of intestinal schistosomiasis.

Our findings indicate significantly higher cure rates of the combination therapy of PZQ and DHP compared to PZQ alone at both post-treatment follow-up visits. At three weeks follow-up visit, the combination therapy produced a significantly higher cure rate of 88.3% compared to 81.2% in the PZQ alone. At eight weeks, there was a significant drop in the cure rates in the PZQ alone group (63.9%) compared to 81.9% in the PZQ and DHP combination group. As observed in this study, PZQ and DHP combination therapy maintained higher cure rates (>80%) at both follow-up visits. The higher cure rate at the 8 week of post treatment in the combination therapy is most likely due to the additional killing effect of DHP on the juvenile worms. Previous randomized clinical trials reported that combination of praziquantel with either artemether or artesunate result in a better outcome than PZQ alone for the treatment and prevention of human schistosomiasis [30]. Previous systematic review and meta-analysis studies concluded that PZQ and artemether or artesunate administrated in combination are more effective than PZQ alone therapy especially to treat patients with repeated exposure to infected water [28, 29].

The higher decline in cure rate from 3 weeks to 8 weeks in the PZQ alone group may indicates that survived juvenile worms attaining reproductive maturity to start laying eggs. Therefore schistosomiasis control and elimination might not be achievable by using mass PZQ administration alone. The observed lower cure rates (63.9%) at eight weeks post-treatment in the PZQ alone group in our study is similar to a recent study conducted to assess the utility of repeated doses of PZQ in an endemic setting along the shores of Lake Victoria in Tanzania, where a cure rate of 68.7% using PZQ single dose at 8 weeks post-treatment was reported [23]. In another study conducted along Lake Victoria in Uganda, much lower cure rate (47.9%) using PZQ alone at 9 weeks post-treatment was reported [48].

Moreover, the combination therapy of PZQ and DHP produced higher cure rates at all levels of infection intensities (light to heavy infections) at both follow-up visits compared to PZQ alone. At 3 weeks follow-up, the combination therapy cure rates were 94%, 89.8% and 80.5% whereas in PZQ alone cure rates were 82.8%, 82.9% and 77.5% for light, moderate and heavy infection, respectively. At 3 weeks follow-up visit, the combination therapy produced significantly higher cure rates than PZQ alone in light infection only. As DHP has little effect against mature parasites, at 3 weeks not much difference between treatment groups would be expected. But at 8 weeks visit, cure rates in the combination therapy were significantly higher (88.0%, 82.0% and 75.9%) compared to that of PZQ alone (63.2%, 67.1% and 59.8%) for light, moderate and heavy infections, respectively. This is an important finding because an intervention that produces a long-term effect in terms of disease cure and control is urgently needed. Therefore, the combination therapy of PZQ and DHP has shown its superiority to PZQ alone in terms of long-term cure rates at all level of infection intensities. A study conducted to assess the efficacy of a combination of PZQ and artesunate in Nigeria for the treatment of urinary schistosomiasis reported similar findings where PZQ and artesunate combination produced higher cure rates in both light and heavy infections compared to PZQ alone [30].

Once more, the combination therapy of PZQ and DHP produced significantly higher ERR of 93.6% compared to PZQ (87.9%) at 8 weeks post-treatment. The reported ERR for PZQ alone (87.9%) at 8 weeks post-treatment falls below the WHO recommended ERR of ≥90% [45]. Egg reduction rates at 3 weeks were almost similar 95.3% in combination therapy group versus 95.0% in the PZQ group. This finding means that the benefits of DHP in killing the immature parasite is observed at a later stage (> 1 month post-treatment) as the direct killing of parasites (both matured and immature) who reside intravascularly cannot be measured [46]. In the PZQ alone group, the observed significant drop in ERR (87.9%) correlates with the observed drop-in cure rates (63.9%) at 8 weeks post-treatment.

The observed low cure rate and ERR of single dose PZQ therapy indicates a need for an alternative treatment strategy for PZQ mass drug administration; first to improve the cure rates thus hastening reduction in morbidity associated with schistosomiasis and second, to control the disease and lastly, to reduce the risk of resistance developing against PZQ [26]. The combination therapy of PZQ and ACTs such as DHP may address the above concerns and can be an option for MDA and use in endemic settings where infected patients harbor more immature worms that are not killed by PZQ. Because of the superior efficacy, DHP plus PZQ combination therapy can be considered as a better option for MDA than single dose PZQ to control and eventually eliminate schistosomiasis. However, the pros and cons of using DHP which is also an antimalarial drug as part of MDA for schistosomiasis treatment and control, particularly in malaria-endemic settings need to be evaluated. Therefore, further researches are needed to assess the feasibility, cost effectiveness and usefulness of this combination therapy of PZQ plus ACTs mass drug administration for treatment and control of schistosomiasis [31, 47].

Interestingly, despite the observed difference in vomiting in small proportion of study participants, the combination of PZQ and DHP was found to be equally safe to the study participants as the standard PZQ alone. The addition of drug(s) to PZQ would be expected to be associated with significant increase in the total proportion of adverse events; however, in the present study, overall there was no significant differences in the occurrence of adverse events between the two treatment groups. Furthermore, almost all adverse events including vomiting were mild and transient in both treatment groups. This finding was important because safety has been maintained even in the presence of DHP. A study done in Nigeria to assess efficacy and safety of a combination of PZQ and artesunate for treatment of urogenital schistosomiasis reported a similar findings [30], where the addition of artesunate to PZQ resulted in no significant increase in the occurrence of adverse events. In the present study, post-treatment abdominal pain was the most common observed adverse event in both treatment groups. Though vomiting was observed in small proportion of participants (3.4%), it was more common in the combination therapy group (5.4%) than PZQ alone group (1.8%) (Table 6). No diarrhea or dizziness was observed among study participants similar to a previous report [30]. However, we recommend future studies to assess adverse events over several days especially in the combination therapy group. As it has been shown in this study, the addition of DHP did not affect the safety and tolerability of PZQ, the combination therapy can be an option for the treatment and control of intestinal schistosomiasis in endemic settings.

This study has some limitation: Although there were no significant differences in the distribution of infection intensity at baseline between the two treatment groups (see Table 1), the slightly higher number of participants with moderate and heavy infections in the PZQ alone group might underestimate the cure rates in this group. Furthermore, assessment of safety was done for the first 4 hours of post-treatment duration, since most adverse events associated with PZQ therapy occurs within the first four hours of drug administration [13, 49, 50]. Although, we found no significant difference in the overall occurrence of early adverse events by adding DHP between the two treatment groups, safety monitoring of PZQ and DHP combination therapy may require a longer study follow-up. Thus, future large sample size and active safety cohort event monitoring studies involving PZQ and DHP combination therapy with a longer post-treatment safety follow-up period is recommended. Nevertheless, our study provides an insight into the high efficacy and early safety profile of the PZQ and DHP combination therapy.

### Conclusions

This study demonstrated that a combination of PZQ and DHP is safe, tolerable and more efficacious compared to PZQ alone for the treatment of intestinal schistosomiasis. The proposed combination therapy can be an option for use in endemic areas with high transmission of schistosomiasis where at any time point the residents are continuously infected and harbor more immature parasites. In addition to schistosomiasis treatment and control, DHP may also provide protection against malaria to the children due to its long duration of post-treatment prophylaxis as malaria continues to be a challenge in this age group in SSA. Further studies to evaluate drug-drug interaction and possible influence of pharmacogenetics variations on drug disposition and treatment outcomes remain vital.

## Supporting information

S1 Checklist Efficacy and Safety of Praziquantel and Dihydroartemisinin Piperaquine Combination for Treatment and Control of Intestinal Schistosomiasis; A Randomized, Non-inferiority Clinical Trial. (DOC)

S1 Protocol Trial and Laboratory Protocol. Efficacy and Safety of Praziquantel combined with Dihydroartemisinin-Piperaquine for the treatment of schistosomiasis; Pharmacokinetics and Pharmacogenetics implications of the drugs combination in Tanzania.
(PDF)

S1 Diagram Consort flow diagram. Study participants screening, allocation to treatment and progress in the trial.
(DOC)

S1 Table Association between baseline characteristics, infection intensity with cure rates within treatment groups at 3 weeks post-treatment follow-up visit. (DOCX)

S2 Table Association between baseline characteristics, infection intensity with cure rates within treatment groups at 8 weeks post-treatment follow-up visit. (DOCX)

S3 Table Geometric mean intensity at baseline and follow up visits (3 weeks and 8 weeks post-treatment). (DOCX)
