# Supplementary material for: Efficacy and safety of praziquantel and dihydroartemisinin piperaquine combination for treatment and control of intestinal schistosomiasis: A randomized, non-inferiority clinical trial
Source: PLoS Negl Trop Dis. 2020 Sep 23;14(9):e0008619. doi: 10.1371/journal.pntd.0008619 (PMC7510991; doi:10.1371/journal.pntd.0008619)
Supplement: S1 Protocol — Efficacy and Safety of Praziquantel combined with Dihydroartemisinin-Piperaquine for the treatment of schistosomiasis; Pharmacokinetics and Pharmacogenetics implications of the drugs combination in Tanzania. (PDF) [file pntd.0008619.s002.pdf]

MUHIMBILI UNIVERSITY OF HEALTH AND ALLIED SCIENCES
SCHOOL OF MEDICINE
DEPARTMENT OF CLINICAL PHARMACOLOGY

EFFICACY AND SAFETY OF PRAZIQUANTEL COMBINED WITH DIHYDROARTEMISININ-PIPERAQUINE FOR THE TREATMENT OF SCHISTOSOMIASIS; PHARMACOKINETICS AND PHARMACOGENETICS IMPLICATIONS OF THE DRUGS COMBINATION IN TANZANIA

PhD Proposal

By

RAJABU HUSSEIN

(MD, MSc in Clinical Pharmacology)

Supervisor:

1. **Professor Eleni Aklillu, PhD., Division of Clinical Pharmacology, Department of Laboratory Medicine, Karolinska University Hospital-Huddinge, Karolinska Institutet, Stockholm, Sweden**

2. **2. Professor Omary OMS, PhD., Department of Clinical Pharmacy and Pharmacology, School of Pharmacy, , MUHAS, Dar es Salaam, Tanzania**

Contents

List of abbreviations/acronyms 4

Abstract 5

1.0 Background information 7

1.1 General introduction 7

1.1.1Schistosomiasis 7

1.1.2 Life cycle of the schistosomes 8

1.1.3 Diagnosis 8

1.1.4 Treatment and control of schistosomiasis 10

1.2 Statement of the problem 12

1.3 Rationale of the study 13

1.4 Research Questions 13

1.5 Objectives 14

1.5.1 Broad objective 14

1.5.2 Specific objectives 14

1.6 Literature review 15

1.6.1 Treatment and control of schistosomiasis 15

1.6.2 Metabolism of PZQ and DHP and possible Drug-drug interactions 16

1.6.3 Pharmacogenetics of PZQ and DHP 17

1.6.4 Schistosomiasis – malaria co-infection 17

2.0 Methodology 18

2.1 Study area 18

2.2 Study design and study population 18

2.3 Study participants selection 18

2.3.1 Inclusion criteria 19

2.3.2 Exclusion criteria 19

2.3.3 Sample size 19

2.4 Intervention 20

2.5 Recruitment, randomization and blinding 21

2.6 Study outcomes 21

2.7 Laboratory methods 22

2.7.1 *S. haematobium* and *S. mansoni* diagnosis 22

2.7.2 Malaria diagnosis 22
2.7.3 Blood sampling, processing and storage 23
2.7.4 Assessment of the Pharmacokinetics of PZQ and PQ 23
2.7.5 Assessment of the Pharmacogenetics of PZQ and DHP 23
2.7.6 Assessment of tolerability of the new intervention (PZQ+DHP) vs. PZQ alone 24
2.8 Statistical methods 24
2.8.1 Data quality and management 24
2.8.2 Data and statistical analysis 25
2.9 Ethical consideration 25
2.10 Study limitations 26
3.0 Activity Plan 26
4.0 Budget .... **Error! Bookmark not defined.**
4.1 Budget justification .... **Error! Bookmark not defined.**
5.0 References 27
Appendix III: Material transfer agreement form 40

List of abbreviations/acronyms

ACT	Artemisinin based Combination Therapy
ALu	Artemether-Lumefantrine
CYP P450	Cytochrome P450
DALYs	Disability Adjusted Life Years
DHA	Dihydroartemisinin
DHP	Dihydroartemisinin-Piperaquine
NMCP	National Malaria Control Program
NTDs	Neglected Tropical Diseases
PQ	Piperaquine
PZQ	Praziquantel
POC CCA	Point of Care Circulating Cathodic Antigen
SP	Sulfadoxine-Pyrimethamine
SSA	Sub-Saharan Africa
TMHIS	Tanzania Malaria HIV/AIDS Indicator Survey
WHO	World Health Organization

Abstract

Background: Despite the use of Praziquantel **(PZQ)** for treatment and control of schistosomiasis as mass drug administration (MDA), the disease remains prevalent among school children and adults in Tanzania. The main reason for this failure is thought to be poor activity of PZQ against immature schistosomes. Artemisinin and its derivatives have been proven to be effective against immature schistosomes. Indeed, recent clinical trials with a combination of PZQ and artemisinin or its derivatives show high efficacy in clearing both mature and immature schistosomes. However, in malaria endemic countries like Tanzania, the use of artemisinin without being combined with long half-life partner drug e.g. lumefantrine or piperaquine, exposes malaria parasites to drug pressure increasing risk of artemisinin resistant *P. falciparum* infections. The use of PZQ plus artemisinin combined with partner drug (ACT) for schistosomiasis offers a better option and the risk of emergence of resistant malaria parasites is reduced. PZQ plus ACT has not been tested in patients with schistosomiasis before, thus its effectiveness and safety need to be investigated. We hypothesize that a combination of PZQ and ACTs is superior to PZQ alone for schistosomiasis treatment and control. On the other hand, patients with schistosomiasis are likely to be co-infected with malaria. As a secondary outcome, we intend to determine malaria treatment outcome using Dihydroartemisinin-piperaquine (DHP) in the presence of PZQ

Aim of the study: To determine the efficacy and safety, pharmacokinetic (PK) interactions and pharmacogenetics of PZQ plus DHP for schistosomiasis treatment among school children. This study also aims at determining the treatment outcome of uncomplicated malaria among children who also had malaria when they were initiated a combination of DHP+PZQ for schistosomiasis.

Methodology: The proposed study will conducted in Mwanza region. This will be a prospective open label randomized non-inferiority trial. The proposed study will involve 270 school children (aged 5-15 years) diagnosed with schistosomiasis (microscopically confirmed urine or stool) with or without malaria (using microscopy). The children will be randomized to receive either a combination of PZQ + DHP (Test) or PZQ alone (control). Study participants will be followed up for a period of 2 months and blood samples will be collected for Pharmacokinetics (PK) studies of PZQ and PQ, pharmacogenetics studies, Complete Blood Count (CBC), Liver Function Test (LFT), Renal Function Test (RFT) and Hemoglobin levels. Urine and stool will be collected for determination of schistosome's eggs intensity. The primary outcome will be

schistosomiasis treatment outcome defined as presence or absence of schistosome's eggs in urine or stool at 1 month and 2 months period after treatment in both arms. The secondary outcomes will include malaria treatment outcome, tolerability of both test and control arms as determined by CBC, LFT and RFT, and PK of PZQ and Piperaquine (PQ). Blood samples will be collected for PK and pharmacogenetics studies of PZQ and DHP and safety profiles as defined by CBC, LFT and RFT

Duration: The proposed study will be conducted for a period of four years (from 2016 to 2020).

1.0 Background information

1.1 General introduction

Neglected tropical diseases (NTDs) such as schistosomiasis remain a burden in Sub-Saharan Africa (SSA), creating a public health concern. NTDs mostly affect communities with low income and Sub-Saharan region remains one of the most affected area (1). The diseases are associated with disabilities, preventing children from school attendance, reducing their cognitive abilities, affecting their academic performance thus affecting their future life socially, politically and economically (1–3). These add a burden to poverty. It has been estimated that, about 534000 deaths and more than 57million Disability Adjusted Life-Years (DALYs) are attributed to the NTDs annually (4). NTDs are linked to almost all Millennium development goals (MDGs); and their control is associated with direct impact on the achievement of the MDGs such as reduction of poverty, improvement in nutrition, water and sanitation and education (3).

1.1.1Schistosomiasis

Prevalence of schistosomiasis remains high in SSA (1,5). The disease is endemic in 76 countries worldwide and it is a second parasitic disease to malaria in SSA, affecting more than 200 million people worldwide, of which more than 80% are found in the SSA (1,5). Schistosomiasis alone causes about 150,000-200,000 deaths annually in SSA (6). In Tanzania, the disease is highly prevalent in the lake zones especially among school children (5). Children are the most affected population because of increased contact with infected water when they play for example when swimming. However, all age groups can be affected due to different water contact activities such as fishing and domestic water uses. In children, the disease causes anaemia, growth stunting, fatigue and diminished physical fitness as well as impaired cognitive development (7,8). In later stages the disease may cause renal failure, hydronephrosis and bladder cancer due to chronic inflammation (7,9). The severity of the morbidity increases when schistosomiasis interacts with other parasitic infections such as malaria (10).

Schistosomiasis is caused by a parasite of genus schistosoma (11). Several schistosoma species exist but *S. haematobium* and *S. mansoni* species are predominant in many areas of SSA (1) with *S. haematobium* taking the lead (10).

Apart from its own morbidity, recent studies have shown that, S. *mansoni* increases susceptibility to malaria by changing the balance of Th1 and Th2 immune response hence affecting the immunological protection of malaria (12,13).

1.1.2 Life cycle of the schistosomes

Schistosomes have two stages of life cycle with two different hosts. A man acquires the infection through contaminated freshwater bodies (lakes, dam and rivers) with an infective larvae stage called cercariae. The cercariae penetrate human skin and develop into immature worms (schistosomulae). The immature schistosomulae are carried by blood to the portal vein of the liver where they develop into mature adult worms and this process takes not less than 8 weeks. Thereafter a pair of matured female and male adults migrates against venous blood from the liver to the perivesical (*S.haematobium*) and mesenterical (*S. mansoni*) venous plexuses. Within these venules, female lay up to several hundred eggs per day for several years. These eggs penetrate the tissue causing inflammation and organ damage, while other eggs reaches the lumen of bladder (*S.haematobium*) or colon (*S. mansoni*) and are excreted in the urine and stool respectively

The excreted eggs hatches in freshwater to release miracidia which penetrate the fresh water snails (intermediate host) multiplying to form an infective stage i.e. cercariae, a process taking 4 to 6 weeks. Infective cercariae are released in water searching for a definitive human host to complete the cycle (Figure 1).

1.1.3 Diagnosis

Diagnosis of schistosomiasis is made by microscopic examination and quantification of schistosomes eggs in urine (*S.haematobium*) or faeces (*S. mansoni*), specifically Kato-Katz for *S. mansoni* and urine filtration for *S.haematobium*. The two tests remains as gold standard methods for schistosomiasis diagnosis in resource limited settings and they will be used in this study. Recently, a point of care circulating cathodic antigen (POC CCA) test has been tested for detection of *S. mansoni* antigen in urine and studies have reported its superiority to Kato-Katz method (14). However the test is less sensitive to *S.haematobium*, making it not cost effective especially in the resource limited settings.

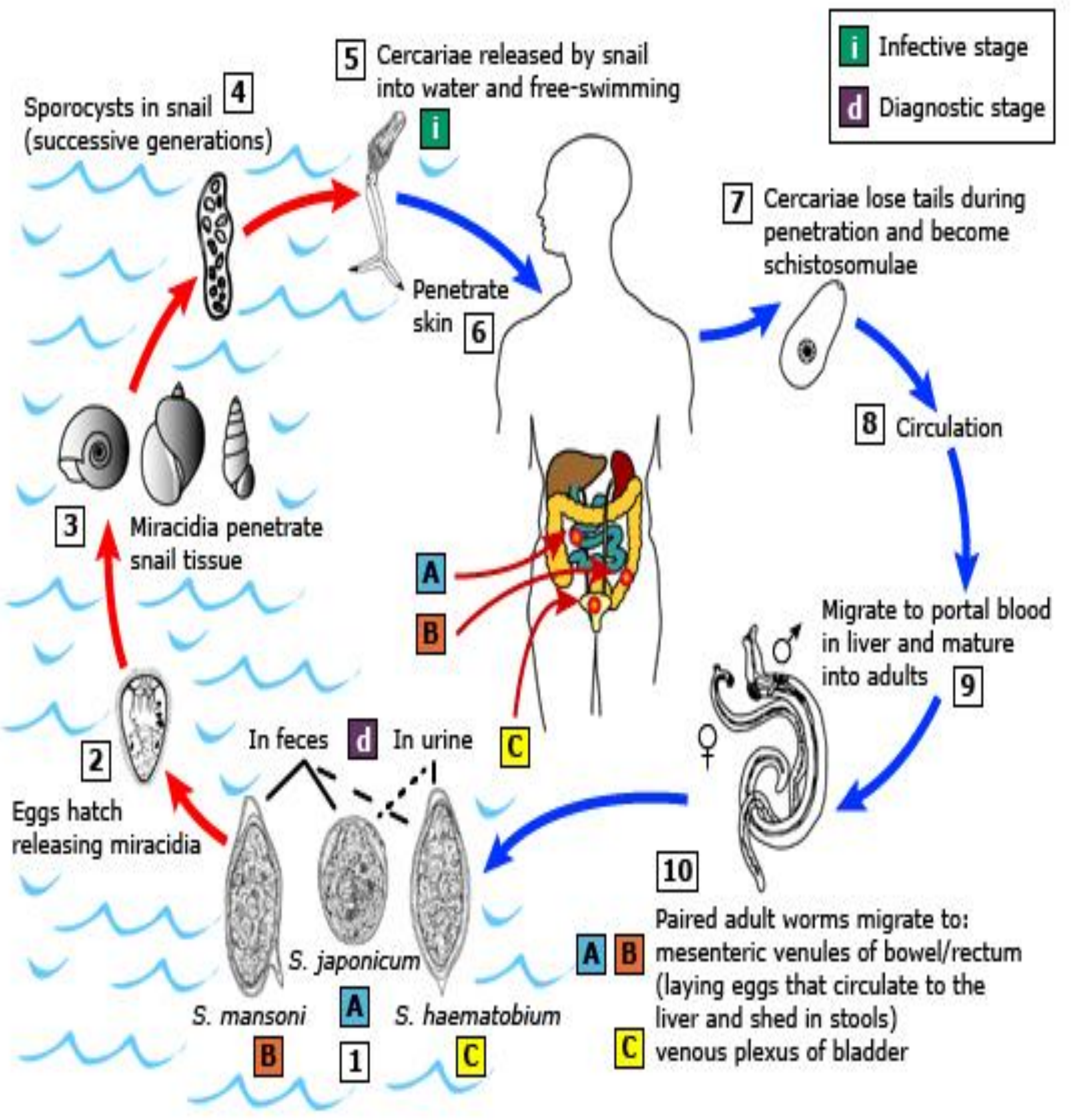

Figure1: Life cycle of S. *mansoni* (Reproduced from: Centers for Disease Control and Prevention; Schistosomiasis. Available at: http://www.cdc.gov/dpdx/schistosomiasis/.)

1.1.4 Treatment and control of schistosomiasis

The WHO promotes five main public health interventions against schistosomiasis. These include preventive chemotherapy, vector control, access to safe drinking water, basic sanitation, and hygiene services and health education. WHO promotes the use of PZQ as preventive chemotherapy and has shown to be cost effective, safe with rapid impact in the control of schistosomiasis in the developing world (15). In Tanzania, mass PZQ was introduced in July 2005 and is given as mass drug administration (MDA) at a dose of 40mg/kg as a single dose for treatment and prophylaxis for schistosomiasis a recommendation of the National NTDs control program

Despite the use of PZQ for mass treatment and disease control, the prevalence of schistosomiasis remains high in endemic settings (5,16). High prevalence reports of this disease in the endemic countries raises a concern on the current efficacy of PZQ. In several studies done in Africa low cure rates with the use of mass PZQ have been reported (17,18).

In general schistosomiasis treatment failure may be attributed by many factors among them being inability of the drug to eliminate a parasite in one or more developmental stages in the life cycle, suboptimal drug concentrations in blood, the use of substandard drug, individual genetics and drug resistance (7,18–21). Since PZQ is sourced from WHO pre-qualified drug companies, it is unlikely that, the available PZQ in Tanzania is sub-standard. Apart from drug quality, suboptimal PZQ concentrations in patients can be attributed by drug-drug interactions and individual's capacity to metabolize the drug. Preliminary data from a recent pharmacokinetics study of PZQ conducted in Ugandan children (3-8 years) who received 40mg/kg as a single dose have shown sub optimal drug plasma concentrations in some children (22). Sporadic studies have reported a threat to PZQ resistance on schistosomes (15, 19, 20) but so far no resistance markers for PZQ has been established.

The main reason for schistosomiasis treatment failure is thought to be poor activity of PZQ against immature schistosomes as PZQ is only effective against mature schistosoma species but has low efficacy against immature schistosomes (juvenile schistosomes) (7). Artemisinin and its derivatives have been proven to be effective against immature schistosomes. Studies have shown encouraging results in clearing the both mature and immature parasites when PZQ is combined with artemisinin or its derivatives (19,20,23) such as artemether. Indeed, Dihydroartemisinin (DHA) which is going to be used in this study has excellent activity against immature

schistosomulae (24).However, in malaria endemic countries like Tanzania, the use of artemisinin (e.g. DHA) for schistosomiasis treatment without being combined with long half-life partner drug e.g. Piperaquine, exposes malaria parasites to drug pressure increasing risk of artemisinin resistant *P. falciparum* infections. The use of PZQ plus artemisinin combined with partner drug (ACT) for schistosomiasis offers a better option as the risk of emergence of resistant malaria parasites is reduced. In fact ACTs given alone (without PZQ) for schistosomiasis treatment have shown very high efficacy(25–27). However, PZQ plus ACTs has not been tested in patients with schistosomiasis before, thus its effectiveness and safety need to be investigated Therefore, in the proposed study we intend to combine PZQ and DHP (as an ACT) for schistosomiasis treatment.
Despite various interventional strategies, *P. falciparum* malaria is still prevalent in Tanzania. Therefore, co-morbidity of schistosomiasis and malaria cannot be underestimated. Co-administration of PZQ plus DHP has not been studied in terms of malaria treatment outcomes since the combination of these drugs poses the risk to drug-drug interactions. PZQ and PQ are all eliminated via CYP450. There is limited information on schistosomiasis or malaria treatment outcomes when both PZQ and DHP are used during co-morbidity.

1.2 Statement of the problem

Schistosomiasis remains a problem in SSA especially in children. Mass PZQ administration has shown insignificant achievement in controlling the prevalence and burden of the disease. Low cure rates of schistosomiasis using mass PZQ administration have been reported in Africa, giving an alarming threat to increased morbidity and mortality rate (28).

Several strategies have been done in attempt to optimize schistosomiasis treatment outcome. For example, Garba *et al* tested the effect of repeated PZQ doses spaced 3 weeks apart but no convincing results were obtained especially in *S. mansoni* infection (29). Olliaro *et al* tested a higher dose of PZQ (60mg/kg) but could not establish significant different results compared to conventional recommended dosing of 40 mg/Kg (30). Similarly, Aloisio *et al* used higher dose of PZQ up to 80mg/kg plus increased treatment duration but could not establish any significant added value rather than increased drug induced toxicity (31)

The main reason for this failure is thought to be poor activity of PZQ against immature schistosomes providing opportunity for the immature parasite to further grow into adults and continue the life cycle (7,9,21) . Recent studies have indicated that, immature schistosomes are sensitive to artemisinin and when combined with PZQ, outstanding treatment outcomes of schistosomiasis in both *S. haematobium* and *S. mansoni* were observed (21, 22,34). The use of PZQ plus artemisinin combined with partner drug (ACT) for schistosomiasis offers a better option and the risk of emergence of resistant malaria parasites is reduced. So far PZQ plus ACT has not been tested in patients with schistosomiasis before and thus, its effectiveness and safety need to be investigated. Therefore, this study will investigate the effectiveness, PK interactions and safety of combining PZQ and DHP for schistosomiasis treatment.

Even when PZQ plus DHP prove to be efficacious in the treatment of schistosomiasis, our second concern will be to prove that the performance of DHP in the treatment of uncomplicated malaria in those patients who took PZQ against schistosomiasis will not be compromised. The two drugs share the same metabolic pathway and drug-drug interaction between PZQ and DHP has not been studied. To our knowledge, information on the treatment outcome of uncomplicated malaria using DHP in patients who just used PZQ for schistosomiasis is lacking.

Furthermore, enzymes involved in the disposition of PZQ and DHP are genetically polymorphic displaying inter-individual variability in enzyme activity, which may affect the PK and PD of PZQ and DHP and may be associated with low cure rates in sub-groups of the population.

Therefore, there is a need to study the genetics of various individuals in Tanzania to determine their effect on the disposition of the two drugs and schistosomiasis treatment outcome.

1.3 Rationale of the study

Findings from this study will provide preliminary data for policy makers especially the Ministry of Health and social welfare in the country on possibility of combining PZQ and ACTs such as DHP in schistosomiasis treatment and control especially in children. The results may also set basis for developing special guidelines in co-treatment of schistosomiasis with malaria. The guidelines will be used by the clinicians to improve quality of care to patients with schistosomiasis and schistosomiasis-malaria co-infection.

1.4 Research Questions

1. Is ACTs-PZQ combination superior to PZQ alone in treatment of schistosomiasis?
2. Is ACTs-PZQ combination well tolerated compared to PZQ alone in the treatment of schistosomiasis?
3. What is the relationship between plasma concentrations of PZQ with the cure rate of schistosomiasis among children?
4. Is there drug interaction of clinical importance between antimalarial (DHP) and antischistosomal drug (PZQ) when used as a combination to treat schistosomiasis?
5. Will malaria treatment outcome using DHP affected in the presence of PZQ?
6. What is the influence of pharmacogenetics variations on the disposition of PZQ and schistosomiasis treatment outcome?

1.5 Objectives

1.5.1 Broad objective

To determine the efficacy and safety of praziquantel combined with Dihydroartemisinin-Piperaquine for the treatment of schistosomiasis; and the pharmacokinetics, pharmacodynamic and pharmacogenetics implications of the drugs combination in Tanzania

1.5.2 Specific objectives

i. To compare the effectiveness and tolerability of PZQ- DHP combination versus PZQ alone for treatment of schistosomiasis among children
ii. To determine the PK profile of PZQ given with DHP in children with schistosomiasis
iii. To determine malaria treatment outcome when DHP is given in the presence of PZQ in children with malaria-schistosomiasis co-infection
iv. To determine the PK profile of PZQ given alone as single dose in children with schistosomiasis
v. To determine the influence of pharmacogenetics of PZQ and DHP in schistosomiasis treatment outcome among children

1.6 Literature review

1.6.1 Treatment and control of schistosomiasis

Mass administration (MDA) of PZQ for treatment and control of schistosomiasis was introduced in Tanzania, in July 2005. Since then the use of drug has scaled up, particularly to the communities with high prevalence of the disease. Mass PZQ is mainly administered to school children but other risk groups in the country are also targeted by the National NTDs control program

Despite PZQ use in schistosomiasis treatment and MDA, the burden of schistosomiasis has continued to be high (5,16,33). One of the reasons that were suspected to cause the PZQ treatment failure is inadequacy of the conventional single dosing in which an individual is given 40mg per body weight. To address this, several studies have been done to investigate the role of higher doses of PZQ for schistosomiasis treatment and control. For example, in a multi centre randomized controlled clinical trial a 60mg/kg of PZQ was tested but no difference in schistosomiasis cure rates were observed compared to conventional recommended dosing of 40 mg/Kg (30). Similarly, in another study a higher dose (up to 80mg/kg) of PZQ plus increasing treatment duration in an attempt to optimize schistosomiasis treatment outcomes but the strategy could not establish any added value instead higher drug induced toxicity were observed (31). Despite that the 40mg/kg body weight of PZQ is associated with low cure rates but still is the recommended dose for schistosomiasis treatment and MDA in Tanzania. This means that an alternative strategy is needed. Under dosing may results into sub-therapeutic PZQ plasma concentration. The proposed study will assess if schistosomiasis patients especially children the most affected population receiving 40mg/kg attain the desired PZQ plasma concentration.

On the other hand, presence of counterfeit PZQ tablets in the market may also explain the reported low cure rates for schistosomiasis. But recently Doenhoff *et al* collected PZQ samples from different producers at users level and it was found that most samples complied well with industrial standards (21). This implies that even in Tanzania the PZQ tablets used for schistosomiasis treatment and control at program level meet the same standards.

Another reason which could also explain this failure is possible emerging PZQ resistance to schistosomes. Sporadic studies have reported a threat to PZQ resistance on schistosomes (15, 19, 20). But no established PZQ resistance has been reported only the parasites were able to survive in drug pressure in laboratory studies.

The main reason for schistosomiasis treatment failure is thought to be inability of PZQ to eliminate a parasite in one of its developmental stages in the life cycle. PZQ is only effective against mature schistosoma species but has low efficacy against immature schistosomes (juvenile schistosomes) (7,9). Thus during MDA, the use of PZQ will only kill mature schistosomes leaving the immature worms unharmed (7). The immature schistosomes will progress into adult stage continuing the life cycle and thus, returning the prevalence and burden of the disease at pre-treatment.

Recent findings indicate that, artemisinin and its derivatives have anti schistosomal activity capable of affecting immature schistosomes (25,27,34,35). For this reason, the use of combination chemotherapy for schistosomiasis treatment and control is highly recommended (36). Randomized controlled clinical trials have shown that a combination of PZQ plus artemisinin or its derivatives clears mature and immature schistosomes and their eggs and in fact result into higher cure rates compared to PZQ alone (20,23,37). DHA which will be used in this study (as DHP) has excellent activity against immature schistosomulae (24). However, in malaria endemic settings like Tanzania, the use of artemisinin (e.g. Artemether, artesunate or DHA) for schistosomiasis treatment without being combined with long half-life partner drug e.g. Lumefantrine or Piperaquine exposes malaria parasites to drug pressure increasing risk of artemisinin resistant *P. falciparum* infections. The use of PZQ plus artemisinin combined with partner drug (ACT) for schistosomiasis offers a better option as the risk of emergence of resistant malaria parasites is reduced. In fact in studies done in African countries where ACTs was given alone (without PZQ) have shown very high efficacy against schistosomes (25–27). However, PZQ plus ACTs has not been tested in patients with schistosomiasis before, thus its effectiveness and safety need to be investigated. Therefore, in the proposed study we intend to combine PZQ and DHP (as an ACT) for schistosomiasis treatment.

1.6.2 Metabolism of PZQ and DHP and possible Drug-drug interactions

However, the presence of more than one drug in the body is associated with a risk of drug-drug interaction. PZQ is as a racemic mixture of R and S enantiomers undergoes extensive first-pass metabolism, is mainly metabolized by CYP3A4. Other enzymes include CYP2C9, and CYP2C19 (38). Dihydroartemisinin is converted to inactive metabolites via glucuronidation catalyzed by UDP-glucuronosyltransferases, in particular UGT1A9 and UGT2B7 (39).But Piperaquine (PQ) a partner drug in DHP is mainly metabolized by CYP3A4 (40) and in fact PQ

has long half life (up to 3 weeks) increasing risk of drug-drug interaction. Since the two drugs share the same metabolic pathway there is a risk of drug interaction. The extent of drug interaction between PZQ and DHP has not been studied. Therefore apart from assessing the efficacy of combining PZQ and DHP, this study will also investigate the PK interactions between the two drugs and determine its effects on schistosomiasis treatment outcomes

1.6.3 Pharmacogenetics of PZQ and DHP

It is well known that drug metabolism via CYP450 enzymes exhibits genetic variability (polymorphism) that may influences treatment outcomes. The enzymes involved in the disposition of PZQ as well as DHP including CYP3A4, CYP 2C9 and CYP 2C19 are genetically polymorphic displaying inter-individual variability in enzyme activity, which may affect the PK and PD of PZQ and DHP and thus, may be associated with low cure rates for schistosomiasis in sub-groups of the population. Therefore, there is a need to study the genetics of various among individuals in Tanzania in order to determine their effect on the disposition of the two drugs and schistosomiasis treatment outcomes.

1.6.4 Schistosomiasis – malaria co-infection

Due to geographical and endemicity overlap in SSA, the co-infection (polyparasitism) between schistosomiasis and malaria is common and region contributes more than 85% of the global burden of the two diseases (25). A study done in Tanzania in 2010 reported the prevalence of schistosomiasis-malaria co-infection as high as 22.6% among school children (10). Several factors facilitate schistosomiasis-malaria co-infection especially in the SSA. These include presence of water bodies (breeding site for both vectors), environmental contamination, poverty and lack of effective preventive measures (41). Co-infection usually results into a prolonged and severe morbidity especially in children. For example in a study done among school children by Mazigo *et al* it was shown that the clinical and pathology presentation of the two diseases becomes more severe in co-infection.(10). Therefore, regular assessment of the drug’s efficacy is required. Schistosomiasis-malaria co-infection is managed by a combination of PZQ and an antimalarial drug usually an ACT. As explained earlier there is a risk of drug-drug interaction when the two drugs are co-administered. This study will also assess malaria treatment outcomes when patients use DHP in the presence of PZQ.

2.0 Methodology

2.1 Study area

The study will be conducted in two areas in the country based on the burden of the disease. In northern zone, the study will be conducted around the shores of Lake Victoria where the disease is highly prevalent(5). Regions such as Mwanza (Magu, Sengerema, Misungwi and Ilemela), Mara (Rorya) and Shinyanga (Bariadi rural/Ikilima) will be included. In the central zone, the study will be conducted in Morogoro (Mvomero, Kilombero/Ifakara and Kilosa). Morogoro is one of the regions which are affected by schistosomiasis (42). The two areas are also highly affected with malaria (43)

2.2 Study design and study population

This will be a prospective open-label randomized clinical non-inferiority trial. School children diagnosed with schistosomiasis tested malaria positive or negative will be enrolled.

2.3 Study participants selection

Multi stage random sampling method will be used to recruit the study participants.

Pre-visit to the study sites will be done before recruitment of study participants. At each district all wards with high prevalence of schistosomiasis will be identified and listed. Using lottery method two wards will be included. From each ward, villages with high prevalence of schistosomiasis will be identified and listed. Using lottery method three villages from each ward will be included. Primary schools available in each village will be identified and listed; using lottery method one primary school will be included into this study. The village leadership and the school authority will be visited to seek for permission to conduct the study.

Screening for schistosomiasis and malaria will be done to all school children available in selected schools during the first month of data collection of this study. The screening methods for both schistosomiasis and malaria are explained in the laboratory methods (page number 22-23). School children who meet the inclusion criteria (Box 1) will be randomly selected to participate in this study. Those who will be schistosomiasis positive or malaria positive and not selected to be part of this study will be referred to the nearby health facility preferably a district hospital for standard treatment. **In addition, participants who will be found with diseases not included in the study will also be referred to the nearby health facility preferably a district hospital for standard treatment**

2.3.1 Inclusion criteria

1. School children aged 5-15 years, both male and females
2. Live primarily in a study village/area
3. School children confirmed schistosomiasis positive by urine filtration test for urinary schistosomiasis and Kato-Katz for intestinal schistosomiasis
4. School children confirmed malaria positive or negative by microscopy
5. Parent/s has/have consented for the child to be recruited in this study
6. The child has assented to participate
7. No known or documented sensitivity to any of the drugs under test

2.3.2 Exclusion criteria

1. Patients without diagnosis of Schistosomiasis (tested negative for schistosomiasis)
2. A recent PZQ treatment before entry to the study (2 months)
3. Presence of danger signs and symptoms of severe malaria according to WHO criteria
4. Prior treatment of malaria within 14 days of study enrollment
5. Patient receiving any medication known to affect cytochrome P 450 within 14 days of study enrollment
6. Pregnant women

Termination criteria

Participants will be excluded from the study if they violate the study protocols. In addition, a patient who develops any severe adverse effect(s) will be discontinued from the study and managed by the study clinician or referred to health care facility for management.

2.3.3 Sample size

The sample size is calculated using the formula

$$n = 2\bar{\pi}(1-\bar{\pi})\frac{(\xi_{1-\alpha/2}+\xi_{1-\beta})^2}{(\pi_c-\pi_t)^2}.$$

Where πc = Cure rate **of** schistosomiasis in the children receiving PZQ alone

πt = Cure rate **of** schistosomiasis in the children receiving PZQ + DHP combination

From a study done by Inyang-Etoh *et al* (19), where efficacy of a combination of **PZQ** and artesunate for treatment of schistosomiasis was tested. The cure rate for **PZQ** alone was 72.7% while that of **PZQ** plus artesunate was 88.7%. We assume the new intervention will have a cure

rate of 90%. Assuming 5% precision, 80% power and 95% significance level, therefore, 80 school children will be recruited in each arm. A total 240 school children will be recruited based on the three arms; which are children with schistosomiasis without malaria receiving PZQ alone (arm 1), children with schistosomiasis without malaria receiving PZQ + DHP (arm 2) and children with schistosomiasis-malaria co-infection receiving PZQ + DHP (arm 3)(Figure 2). Adding 10% of those who will be lost to follow the overall minimum sample size will be **270** school children.

2.4 Intervention

Study participants will have their intervention allocation at enrollment. School children diagnosed with schistosomiasis without malaria will receive either PZQ alone (40mg/kg single dose) or a combination of PZQ + DHP (40mg Dihydroartemisinin / 320mg piperaquine, number of tablets/body weight as directed by the National Malaria Treatment Guidelines). These will be randomized using a lottery method to receive PZQ alone or PZQ + DHP. While school children diagnosed with both schistosomiasis and malaria will receive a combination of PZQ + DHP.

PZQ and first dose of DHP will be given as a direct observed treatment (DOT) and all of the remaining DHP doses will also be given as DOT to rule out inconsistence in drug intake and timing. All participants will be given milk to ensure standardization of drugs absorption.

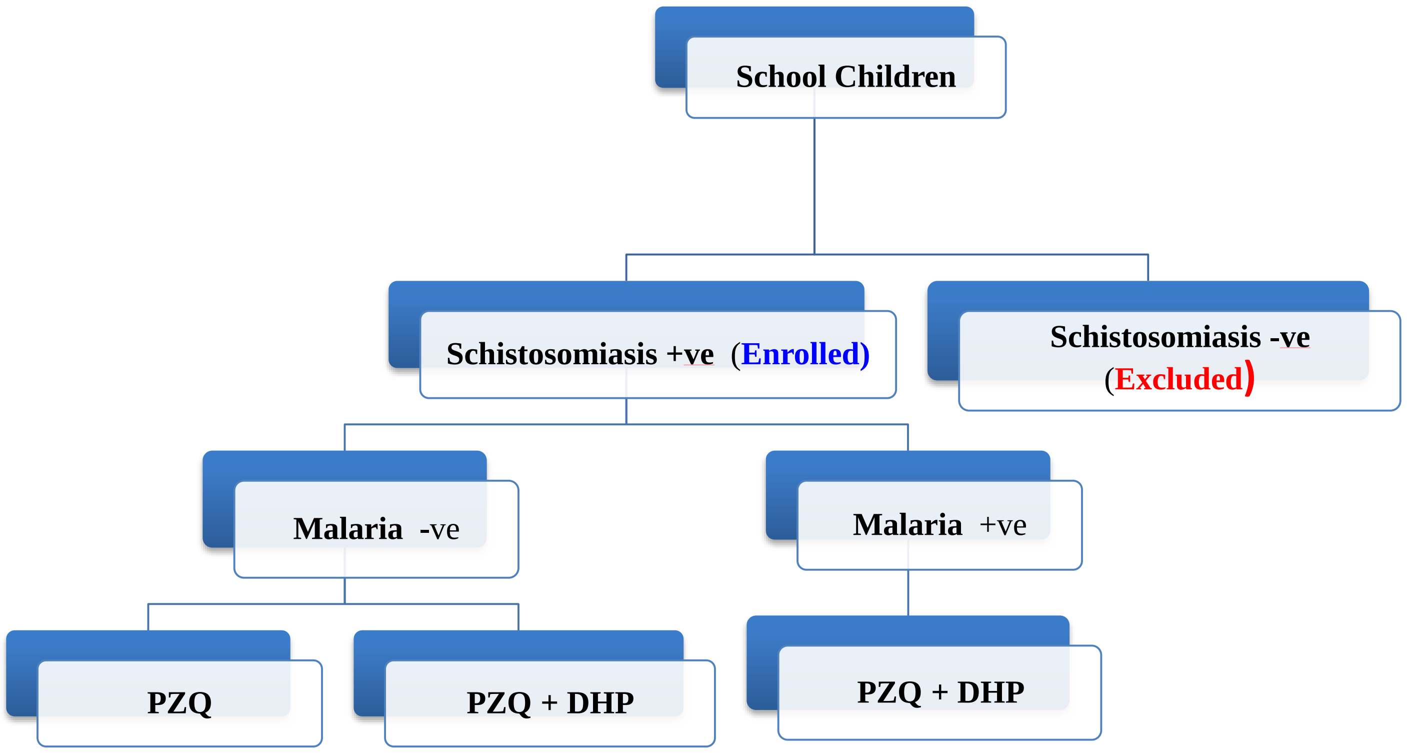

Figure 2: showing the study arms

2.5 Recruitment, randomization and blinding

Participants who will meet the inclusion criteria and consented will be enrolled in the study. Participants will be randomized using a lottery method to receive PZQ alone or PZQ + DHP. Since this study will be an open-label, study participants, study coordinator and investigators will be aware of the treatment allocation.

Follow up period

The school children will be followed up for a period of 2 months from day of enrollment

2.6 Study outcomes

2.6.1 Primary outcome:

- Schistosomiasis treatment outcome defined as presence or absence of eggs in urine or stool at 1 month and 2 months period between the new intervention (PZQ + DHP) and PZQ alone arms

2.6.2 Secondary outcomes:

- DHP and PZQ interaction
- Tolerability of the new intervention (adverse events)
- Malaria treatment outcome (defined as presence or absence of malaria parasite after treatment/parasite clearance)
- PK – PZQ plasma levels
- PK – PQ plasma levels
- Pharmacogenetics of PZQ and DHP for the presence of single nucleotide polymorphism (SNPs)
- Clinical observations and blood biochemistry, including monitoring hemoglobin and alanine aminotransferase levels over time

2.7 Laboratory methods

2.7.1 *S. haematobium* and *S. mansoni* diagnosis

Urine and stool samples will be collected from participants for *S. haematobium* and *S. mansoni* examination. The samples will be examined for the presence of schistosoma eggs using the standard urine filtration technique for *S. haematobium* and Kato-Katz technique for *S. mansoni* for 2 consecutive days (to improve sensitivity of a test). The intensity of schistosoma infection will be categorized based on the WHO cut of value (light infection= 1-99 egg per gram of faeces (epg), moderate infection =100-399 epg and heavy more than 400 epg for *S. mansoni* and <50eggs/10mls of urine=light and >or equal 50eggs/10mls of urine=heavy for *S. haematobium*). After 1 month and then 2 months of treatment, urine and stool samples will be collected again and examined for 2 consecutive days for the presence or absence of schistosoma eggs (the reading will be done by two separate qualified laboratory technician (to improve accuracy), in case of discrepancy a third technician will be involved and a consensus will be reached.

2.7.2 Malaria diagnosis

Finger prick blood samples will be obtained for malaria diagnosis under light microscopy. Both thick and thin blood smears will be prepared from each patient on day zero. Thin blood films will be fixed in absolute methanol for 2 minutes and the two smears (thick and thin) air-dried and stained with 10% standard buffered Giemsa stain (pH 7.2) for 10 minutes. Thick blood smears

will be scanned for the presence of malaria parasite under oil immersion. On the other hand, thin blood smears will be used to confirm *P. falciparum* species. Parasite density will be expressed per 200 white blood cells. Blood samples for examination of malaria parasite will be collected at baseline (day 0), day 3, day 7, day 14 and day 28

2.7.3 Blood sampling, processing and storage

Prior to sample collection, the following data will be collected; date, participant study number and sampling time at which blood is collected. Blood samples (5mls) will be collected after the patient has been found eligible and consented to participate in the study.

Blood samples for clinical chemistry such as LFT (Alanine amino transferase (ALAT) and Aspartate amino transferase (ASAT)), RFT (serum creatinine) and complete blood count will be collected at baseline (day 0), day 3 and 1 months follow up.

Blood sampling for drugs assays will be done using time intervals as specified in the confidential case report form (appendix II).

An aliquot of the collected blood (2mls) will be processed in the laboratory, where centrifugation (x2, 000g for 10 mins) will be done to obtain plasma, which will be transferred to labeled cry vials. The plasma will be kept in the freezer at -80 °C before being transported in cool box to MUHAS-Sida bioanalytical laboratory for analysis

2.7.4 Assessment of the Pharmacokinetics of PZQ and PQ

Analysis of the drugs will be done at bioanalytical laboratory in MUHAS.

- PZQ will be measured by HPLC with UV detection adopting the method described by Hanpitakpong *et al* (44).
- PQ plasma concentration will be measured by HPLC adopting the method described by Hung *et al* (45)

2.7.5 Assessment of the Pharmacogenetics of PZQ and DHP

For a pharmacogenetics study analysis two mls of venous blood will be collected from all participants during enrollment and kept at -80 °C. Blood samples will be shipped on a dry ice to Karolinska Institutet in Sweden for analysis under material transfer regulation (appendix III). Genomic DNA will be isolated using a QiAmp DNA Mini Kit (Qiagen GmbH, Germany). Genotyping for known functional variant alleles in gene coding for drug metabolizing enzymes

e.g. CYP 3A4 relevant for metabolism of PZQ and piperaquine will be done at Karolinska Institutet in Sweden according to the TaqMan drug metabolism genotyping assay method for allelic discrimination (Applied Biosystems Genotyping Assays) previously described by Shi *et al* (46)

2.7.6 Assessment of tolerability of the new intervention (PZQ+DHP) vs. PZQ alone

A structured checklist (Appendix II) will be used to record the adverse events from both arms. The changes observed from baseline condition will be regarded as adverse events

Tolerability will also be determined by tests such as LFT, RFT and CBC at baseline (day 0), day 3 and at1 month follow up.

In each site, a clinical officer and nurse will be added as part of the study team to assist the PI to assess the adverse events from participants

2.8 Statistical methods

2.8.1 Data quality and management

A PhD student will visit the study site regularly to recruit participants, ensure that the standard operating procedures (SOP) for screening of the participants, blood sample collection and storage are followed. During laboratory examination of stool and urine specimens, a 10% sample of specimens will be re-examined by an independent technician (one who is not part of the research team) every day and results compared to see the degree of agreement.

Data will be kept in a password secured computer to ensure confidentiality and all stool, urine, blood samples and documents during the study period will be stored by the PI until time for destruction after the data have been analyzed and published.

Data and Safety monitoring board (DSMB) will be established at study initiation to monitor severe adverse events (SAEs) and to approve the statistical analysis plan and associated stopping rules for benefits or harm. The DSMB will include expertise in clinical trial, statistics and ethics. The initial meeting will be held before patient recruitment is initiated

2.8.2 Data and statistical analysis

The data will be double entered into a computer by a data entry clerk and the entered data will then be verified and cleaned before being subjected to analysis using the different statistical programs e.g. SPSS, and STATA. Intention to treat analysis will be done

Chi- square or Fisher's exact test will be used for comparison of proportions (eg. cure rate & egg reduction rate, frequency of AEs and severity of AEs) and Student's t test for comparison of means (geometric mean egg output, mean drugs plasma concentration) in the two treatment groups (PZQ 40mg/kg alone and PZQ-DHP combination therapy).

Descriptive statistics will also be used where appropriate. A two tailed P-value of <0.05 will be considered significant

2.9 Ethical consideration

Ethical clearance will be requested from MUHAS Ethical commitee or Institutional Review Board (IRB), National Institute of Medical Research (NIMR) ethical commitee and Karolinska Institutet (KI) ethical commitee before commencement of the study within the proposed study areas. The permission letters will be sent to the concerned authority prior data collection.

At the beginning of the study meetings will be organized with parents and teachers of the earmarked schools, whereby detailed information will be provided by the research team about the aims, procedures, benefits and potential risks of the study. Written informed consent will be obtained from all participants. In case of minors, the oral assent of the child will be asked, besides the written consent of the legally acceptable representative (LAR)/guardian. LAR/guardian who is illiterate will be asked for verbal consent in front of an independent literate witness who will be asked to countersign the consent form on his/her behalf. If the child is between 6 - <13, and according to the investigator perception, he/she is not able to understand the information given during informed consent procedure, the oral assent is not necessary. Participation in the study will be on voluntary terms and that an individual will be free to withdraw from the study at any time. Withdrawal from the study will not affect ones statutory rights in any

All records of study subjects will be kept confidential and will be kept locked in a cabinet. Only one designated person (the PI) of the team will be responsible for them, and no other study team members will access them. A unique identification number will be provided to consented

participants such that the information collected during the study will not reveal the identity of the participant to maintain confidentiality**.**

Small amount of venous blood sample will be taken from the participants, we will not subject participants to any complications as a result of blood withdrawal (chance for complications is very minimal).

All information obtained will be treated as confidential and will be used for the purpose of the study only. No names of study participants will be used and instead only code number will be used during data collection and analysis. The information generated in this study will be of beneficial to the participants, the community around and country as whole

2.10 Study limitations

- Adherence to the three doses of ACT i.e. DHP for 3 days could be a problem

2.11 Mitigation measures

- Education will be given to all parents and participants on the importance of completing the treatment given, 1st dose will be taken as DOT. Moreover, patients will be asked to bring the empty blisters to the clinic to ensure compliance to medications

2.12 Dissemination plan

The study findings will be disseminated by:

1. Publications in peer review journals
2. Presentation at International, regional and local scientific conferences
3. Public defense of the thesis at University level
4. Presentation at the Ministry of Health/ policy makers, National Institute of Medical research/NTDs section
5. Official meeting with the study participants, community around study site and their leaders.

5.0 References

1. Adenowo AF, Oyinloye BE, Ogunyinka BI, Kappo AP. Impact of human schistosomiasis in sub-Saharan Africa. Brazilian J Infect Dis [Internet]. Elsevier Editora Ltda; 2015;19(2):196–205. Available from: http://dx.doi.org/10.1016/j.bjid.2014.11.004
2. Gray DJ, Ross AG, Li Y-S, McManus DP. Diagnosis and management of schistosomiasis. BMJ. 2011;342(May):d2651.
3. Samuels F, Pose RR. Why neglected tropical diseases matter in reducing poverty. Dev Prog. 2013;(July).
4. Hotez PJ, Molyneux DH, Fenwick A, Ottesen E, Ehrlich Sachs S, Sachs JD. Incorporating a Rapid-Impact Package for Neglected Tropical Diseases with Programs for HIV/AIDS, Tuberculosis, and Malaria. PLoS Med [Internet]. 2006;3(5):e102. Available from: http://www.plosmedicine.org/article/info:doi/10.1371/journal.pmed.0030102
5. Mazigo HD, Nuwaha F, Kinung'hi SM, Morona D, de Moira AP, Wilson S, et al. Epidemiology and control of human schistosomiasis in Tanzania. Parasit Vectors [Internet]. Parasites & Vectors; 2012;5(1):274. Available from: http://www.parasitesandvectors.com/content/5/1/274
6. Hotez PJ, Fenwick A, Savioli L, Molyneux DH. Rescuing the bottom billion through control of neglected tropical diseases. Lancet [Internet]. Elsevier Ltd; 2009;373(9674):1570–5. Available from: http://dx.doi.org/10.1016/S0140-6736(09)60233-6
7. Inobaya MT, Olveda RM, Chau TNP, OlvedoDU and RA. Prevention and control of schistosomiasis: a current perspective. Res Rep Trop Med. 2014;5(1):70–6.
8. Kinung'hi SM, Magnussen P, Kishamawe C, Todd J, Vennervald BJ. The impact of anthelmintic treatment intervention on malaria infection and anaemia in school and preschool children in Magu district, Tanzania: an open label randomised intervention trial. BMC Infect Dis [Internet]. 2015;15(1):136. Available from: http://www.pubmedcentral.nih.gov/articlerender.fcgi?artid=4391149&tool=pmcentrez&rendertype=abstract
9. WHO Technical Report Series No 972. Reasearch Priorities for helminth infections: Techincal report of the TDR disease reference group on Helminth Infections. 2012.
10. Mazigo HD, Waihenya R, Lwambo NJ, Mnyone LL, Mahande AM, Seni J, et al. Co-

infections with Plasmodium falciparum, Schistosoma mansoni and intestinal helminths among schoolchildren in endemic areas of northwestern Tanzania. Parasit Vectors. 2010;3:44.

11. Deribew K, Petros B. Efficacy of praziquantel for the treatment of schistosomiasis in Ethiopia. Int J Med Med Sci. 2013;5(March):131–9.

12. Downs JA, van Dam GJ, Changalucha JM, Corstjens PLAM, Peck RN, de Dood CJ, et al. Association of Schistosomiasis and HIV Infection in Tanzania. Am J Trop Med Hyg [Internet]. 2012;87(5):868–73. Available from: http://www.ajtmh.org/cgi/doi/10.4269/ajtmh.2012.12-0395

13. Ndeffo Mbah ML, Poolman EM, Drain PK, Coffee MP, van der Werf MJ, Galvani AP. HIV and Schistosoma haematobium prevalences correlate in sub-Saharan Africa. Trop Med Int Heal. 2013;18(00):1174–9.

14. Becker SL, Marti H, Zimmermann S, Vidacek D, Herrmann M, Utzinger J, et al. Application in Europe of a urine-based rapid diagnostic test for confirmation of Schistosoma mansoni infection in migrants from endemic areas. Euro Surveill Bull Eur sur les Mal Transm = Eur Commun Dis Bull. 2015;20(23):1–5.

15. Fenwick A, Webster JP, Bosque-Oliva E, Blair L, Fleming FM, Zhang Y, et al. The Schistosomiasis Control Initiative (SCI): rationale, development and implementation from 2002-2008. Parasitology [Internet]. 2009;136(13):1719–30. Available from: http://www.ncbi.nlm.nih.gov/pubmed/19631008

16. Mazigo HD, Waihenya R, Mkoji GM, Zinga M, Ambrose EE, Jahanpour OF, et al. Intestinal Schistosomiasis : Prevalence , Knowledge , Attitude and Practices Among School Children in an Endemic Area of North Western Tanzania. J Rural Trop Public Heal. 2010;9:53–60.

17. Gryseels B, Mbaye A, De Vlas SJ, Stelma FF, Guissé F, Van Lieshout L, et al. Are poor responses to praziquantel for the treatment of Schistosoma mansoni infections in Senegal due to resistance? An overview of the evidence. Trop Med Int Heal. 2001;6(11):864–73.

18. Muheki ET. The effect of one versus two Praziquantel treatments on Shistosoma mansoni morbidity and re-infection along Lake Victoria in Uganda. a thesis degree Dr Philos Makerere Univ. 2011;(February).

19. Inyang-Etoh PC, Ejezie GC, Useh MF, Inyang-Etoh EC. Efficacy of a combination of

praziquantel and artesunate in the treatment of urinary schistosomiasis in Nigeria. Trans R Soc Trop Med Hyg. 2009;103(1):38–44.

20. Liu R, Dong H-F, Guo Y, Zhao Q-P, Jiang M-S. Efficacy of praziquantel and artemisinin derivatives for the treatment and prevention of human schistosomiasis: a systematic review and meta-analysis. Parasit Vectors [Internet]. BioMed Central Ltd; 2011;4(1):201. Available from: http://www.parasitesandvectors.com/content/4/1/201
21. Doenhoff J, Cioli D, Utzinger J. Praziquantel: mechanisms of action, resistance and new derivatives for schistosomiasis. Wolters Kluwer Heal. 2008;21:659–67.
22. Workshop Welcome Booklet. Int Sci Work Neglected Trop Dis Magaliesburg, South Africa. 2015;25.
23. Perez del Villar L, Burguillo FJ, Lopez-Aban J, Muro A. Systematic Review and Meta-Analysis of Artemisinin Based Therapies for the Treatment and Prevention of Schistosomiasis. PLoS One. 2012;7(9).
24. Li H, Xu F, Wang Y. Dihydroartemisinin : a new story of an old drug against Schistosoma mansoni infection. 2014;239–41.
25. Abay SM, Tilahun M, Fikrie N, Habtewold A. Plasmodium falciparum and Schistosoma mansoni coinfection and the side benefit of artemether-lumefantrine in malaria patients. J Infect Dev Ctries. 2013;7(6):468–74.
26. Adam I, Elhardello OA, Elhadi MO, Abdalla E, Elmardi KA, Jansen FH. The antischistosomal efficacies of artesunate-sulfamethoxypyrazine-pyrimethamine and artemether-lumefantrine administered as treatment for uncomplicated, Plasmodium falciparum malaria. Ann Trop Med Parasitol. 2008;102(1):39–44.
27. Adedoja AA, Akanbi AA, Oshodi AJ. Effect of artemether-lumefantrine treatment of falciparum malaria on urogenital schistosomiasis in co-infected School Aged Children in North Central of Nigeria. intJBiolChemSci. 2015;9(February):134–40.
28. WHO. Working to overcome the global impact of neglected tropical diseases. 2010;129.
29. Garba A, Lamine MS, Barkiré N, Djibo A, Sofo B, Gouvras AN, et al. Efficacy and safety of two closely spaced doses of praziquantel against Schistosoma haematobium and S. mansoni and re-infection patterns in school-aged children in Niger. Acta Trop [Internet]. Elsevier B.V.; 2013;128(2):334–44. Available from: http://dx.doi.org/10.1016/j.actatropica.2012.08.008

30. Olliaro PL, Vaillant MT, Belizario VJ, Lwambo NJS, Ouldabdallahi M, Pieri OS, et al. A multicentre randomized controlled trial of the efficacy and safety of single-dose praziquantel at 40 mg/kg vs. 60 mg/kg for treating intestinal schistosomiasis in the Philippines, Mauritania, Tanzania and Brazil. PLoS Negl Trop Dis. 2011;5(6).
31. Aloisio Sales da Cunha RC and GL de R. Therapeutical Evaluation of Different Dose Regimens of Praziouantel in schistosomiasis mansoni, based on the quantitative oogram technique. Rev Inst Med trop. 1987;29(5):295–304.
32. Liu R, Dong H-F, Guo Y, Zhao Q-P, Jiang M-S. Efficacy of praziquantel and artemisinin derivatives for the treatment and prevention of human schistosomiasis: a systematic review and meta-analysis. Parasit Vectors [Internet]. 2011;4(1):201. Available from: http://www.parasitesandvectors.com/content/4/1/201
33. Siza JE, Kaatano GM, Chai J-Y, Eom KS, Rim H-J, Yong T-S, et al. Prevalence of Schistosomes and Soil-Transmitted Helminths and Morbidity Associated with Schistosomiasis among Adult Population in Lake Victoria Basin, Tanzania. Korean J Parasitol [Internet]. 2015;53(5):525–33. Available from: http://www.ncbi.nlm.nih.gov/pubmed/26537031
34. Keiser J, N'Guessan NA, Adoubryn KD, Silué KD, Vounatsou P, Hatz C, et al. Efficacy and Safety of Mefloquine, Artesunate, Mefloquine-Artesunate, and Praziquantel against Schistosoma haematobium : Randomized, Exploratory Open Label Trial. Clin Infect Dis [Internet]. 2010;50(9):1205–13. Available from: http://cid.oxfordjournals.org/lookup/doi/10.1086/651682
35. Boulanger D, Dieng Y, Cisse B, Remoue F, Capuano F, Dieme JL, et al. Antischistosomal efficacy of artesunate combination therapies administered as curative treatments for malaria attacks. Trans R Soc Trop Med Hyg. 2007;101(2):113–6.
36. Jurg U, Keiser J, Shuhua X, Shuhua X, Tanner M, Tanner M, et al. Combination Chemotherapy of Schistosomiasis in Laboratory Studies and Clinical Trials. Microbiology. 2003;47(5):1487–95.
37. Utzinger J, Shuhua X, N'Goran EK, Bergquist R, Tanner M. The potential of artemether for the control of schistosomiasis. Int J Parasitol. 2001;31(14):1549–62.
38. Wang H, Fang ZZ, ZhengY, Zhou K, Hu C, Krausz, Sun D, Idle JR GF. Metabolic profiling of praziquantel enantiomers. Biochem Pharmacol. 2015;90(2):166–78.

39. Ilett KF, Ethell BT, Maggs JL, Davis TME, Batty KT, Burchell B, et al. Glucuronidation of dihydroartemisinin in vivo and by human liver microsome and expressed UDP-glucuronosyltransferases. Drug Metab Dispos. 2002;30(9):1005–12.
40. Lee TM-N, Huang L, Johnson MK, Lizak P, Kroetz D, Aweeka F, et al. In vitro metabolism of piperaquine is primarily mediated by CYP3A4. Xenobiotica. 2012;42(May):1088–95.
41. Booth M. The role of residential location in apparent helminth and malaria associations. Trends Parasitol. 2006;22(8):359–62.
42. Zumstein A. A study of some factors influencing the epidemiology of urinary schistosomiasis at Ifakara (Kilombero District, Morogoro Region, Tanzania). Acta Trop [Internet]. 1983;40(3):187–204. Available from: http://europepmc.org/abstract/med/6138972
43. National Bureauof Statistics. Tanzania Demographic and Health Survey and Malaria Indicator Survey, 2015-16.
44. Hanpitakpong W, Banmairuroi V, Kamanikom B, Choemung A, Na-bangchang K. A high-performance liquid chromatographic method for determination of praziquantel in plasma. J Pharm Biomed Anal. 2004;36:871–6.
45. Hung TY, Davis TME, Ilett KF. Measurement of piperaquine in plasma by liquid chromatography with ultraviolet absorbance detection. J Chromatogr B Anal Technol Biomed Life Sci. 2003;791(1-2):93–101.
46. Shi MM, Myrand SP, Bleavins MR, de la Iglesia F a. High throughput genotyping for the detection of a single nucleotide polymorphism in NAD(P)H quinone oxidoreductase (DT diaphorase) using TaqMan probes. Mol Pathol [Internet]. 1999;52(5):295–9. Available from: http://www.pubmedcentral.nih.gov/articlerender.fcgi?artid=395713&tool=pmcentrez&rendertype=abstract

Appendix II: Confidential case record forms (CRF)

PROJECT: EFFICACY AND SAFETY OF PRAZIQUANTEL COMBINED WITH DIHYDROARTEMISININ-PIPERAQUINE FOR THE TREATMENT OF SCHISTOSOMIASIS; PHARMACOKINETICS, PHARMACODYNAMICS AND PHARMACOGENETICS IMPLICATIONS OF THE DRUGS COMBINATION IN TANZANIA

To be filled on Day 0

PARTICIPANT INFORMATION AND MEDICAL HISTORY

INFORMATIONS:

Date ____________________

Patient ID____________________

Name of school ____________________

Address ____________________

Mobile No (parent/caretaker) ____________________

Next of kin mobile number ____________________

Demographics

Date of birth __________ Age ______________

Sex Male ☐ Female ☐

Schistosomiasis screening

S. mansoni +ve ☐ -ve ☐

Number of eggs per gram of stool ____________________

S. haematobium +ve ☐ -ve ☐

Number of eggs per gram of stool ____________________

Malaria parasite screening

Microscopic test +ve ☐ -ve ☐

Number of parasite per 200 leucocytes ____________________

Drug history

Any other drug used by the participant Yes ☐ No ☐

If yes specify

Drug name	Starting date	End date	Possibility of interaction with PZQ or ACT

Any history of using PZQ in the past 2 months? Yes ☐ No ☐

Any history of using antimalarial (ACT) in the past 2 weeks (14 days) Yes ☐ No ☐

Dou you smoke? Yes ☐ No ☐

Do you drink coffee? Yes ☐ No ☐

Pre dose blood sampling

Drug administration and blood sampling

Date ______________________

Patient ID ______________________

Drug	Dose	Time of drug administration	Time of blood sampling

Blood sampling for PZQ population pharmacokinetics in school children

Real sampling tine (hrs)		
1hr		
2hrs		
3hrs		
4hrs		
6hrs		
12hrs		
24hrs		

Blood sampling for interaction study (PZQ + DHP) in school children

Real sampling time	Amount collected (in mls)	
	PZQ	PQ

Adverse events (observed)
Tick (√) where appropriate
Fever ☐
Nausea ☐
Abdominal pain ☐
Dizziness ☐
Headache ☐
Other specify ☐

**Test**		**Results**	**Remarks**
**LFT**	**ALAT**		
	**ASAT**		
**RFT**	**Serum creatinine**		
**CBC**	**Hb level**		

ADHERENCE AND DRUG MONITORING DAY 3/7 (SWAHILI VERSION)

NAMBA YA HOSPITALI: ________________

NAMBA YA MGONJWA: _________________

1. Baadhi ya watu wanaona vigumu kumeza vidonge vya dawa za malaria (DHP) kila mara. Je toka uanza kutumia dawa hizo kuna vidonge vyovyote ulivosahau kumeza kama ulivoshauriwa na muhudumu wa afya?

   Ndio ☐ Hapana ☐

2. Kama ndio umesahau kumeza dozi ngapi?
   Moja ☐
   Mbili ☐
3. Je ni sababu zipi zinaweza kuwa zimesababisha usahau kumeza dawa mseto kwa matibabu ya malaria?
   (a) Mambo mengi ☐
   (b) Nilisahau tu. ☐
   (c) Nilikuwa mbali na nyumbani ☐

☐

(d) Mabadiliko katika shuguli zangu za kila siku
(e) Sikujisikia ☐
(f) Vidonge vingi ☐
(g) Niliona dawa hii ni sumu ☐
(h) Nilijisikia vibaya baada ya kunywa dawa hii ☐
(i) Nilichanganyikiwa na maelezo ya jinsi ya kutumia dawa hii ☐
(j) Watu waliniambia dawa hii sio nzuri ☐
(k) Taja sababu ingine ____

4. Ulipatiwa maziwa kwaajili ya kumezea dawa hizo, Je kuna dozi yoyote ambayo hukumezea maziwa?
   Ndio ☐ Hapana ☐
5. Kama ndio, ni dozi ipi hukumezea maziwa?
   Dozi za siku ya pili ☐
   Dozi za siku ya tatu ☐

To be filled on Day 28 (1 month after treatment)

To be filled for those who are malaria negative

PZQ alone arm

Date ___________________

Patient ID ___________________

Schistosomiasis screening

S. mansoni +ve -ve

Number of eggs per gram of stool ________________________

S. haematobium +ve ☐ -ve ☐

Number of eggs per gram of stool ________________________

PZQ + ACT arm

Date ________________

Patient ID ________________

Schistosomiasis screening

S. mansoni +ve -ve

Number of eggs per gram of stool ________________________

S. haematobium +ve ☐ -ve ☐

Number of eggs per gram of stool ________________________

To be filled to those who are schistosomiasis malaria co infected

Date ______________

Patient ID ______________

Schistosomiasis screening

S. mansoni +ve -ve

Number of eggs per gram of stool ______________________

S. haematobium +ve ☐ -ve ☐

Number of eggs per gram of stool ______________________

Malaria screening

Microscopic test +ve ☐ -ve ☐

Number of parasite per 200 leucocytes ______________________

To be filled at day 56 (2 months after treatment)

To be filled for those who are malaria negative

PZQ alone arm

Date ______________

Patient ID ______________

Schistosomiasis screening

S. mansoni +ve -ve

Number of eggs per gram of stool ______________________

S. haematobium +ve ☐ -ve ☐

Number of eggs per gram of stool ______________________

PZQ + ACT arm

Date ________________

Patient ID ________________

Schistosomiasis screening

S. mansoni +ve -ve

Number of eggs per gram of stool ___________________________

S. haematobium +ve ☐ -ve ☐

Number of eggs per gram of stool ___________________________

To be filled to those who are schistosomiasis malaria co infected

Date ________________

Patient ID ________________

Schistosomiasis screening

S. mansoni +ve -ve

Number of eggs per gram of stool ___________________________

S. haematobium +ve ☐ -ve ☐

Number of eggs per gram of stool ___________________________

Malaria screening

Microscopic test +ve ☐ -ve ☐

Number of parasite per 200 leucocytes ___________________________

STUDY EXIT FORM

Date ______________ Patient ID ______________

1. Has the participant completed the study? Yes ☐ No ☐
2. Schistosomiasis treatment effective Yes ☐ No ☐
3. Malaria treatment effective Yes ☐ No ☐
4. Indicate the primary reason why the subject did not completed the study
    i. Adverse event ☐
    ii. Protocol violation ☐
    iii. Subject request ☐
    iv. Lost to follow up ☐
    v. Premature study closure ☐
    vi. Other specify ______________

Appendix III: Material transfer agreement form:

1. This Material Transfer Agreement is made and entered as of the ______ day of __________, 20______ (the "Effective Date") by and between MUHIMBILI UNIVERSITY OF HEALTH AND ALLIED SCIENCES and referred to as MUHAS, (hereinafter referred to as "PROVIDER") having its principle office at MUHIMBILI, DAR-ES-SALAAM,TANZANIA and ______________________________________ (Hereinafter referred to a "RECEIVER") having its principle office at ____.

2. In consideration of the mutual covenants contained herein and with the intention of being legally bound under the National and International guidelines rules and regulations applicable to the Research Project and the handling of the RESEARCH MATERIAL :This research material and its derivatives will be used by recipient's investigator solely in connection with the following research project ("Research project") described with specificity as follows ***"Efficacy and safety of Praziquantel combined with Dihydroartemisinin-Piperaquine for the treatment of schistosomiasis; pharmacokinetics, pharmacodynamics and pharmacogenetics implications of the drugs combination in Tanzania"***

3. *"* The "MATERIAL" (hereinafter referred to as "RESEARCH MATERIAL") covered by this Agreement is defined as biological research material and Includes the following:
   ____
   ____
   ____
   ____ developed by ____.

4. RECEIVER desires to obtain samples of the RESEARCH MATERIAL and the PROVIDER is willing to provide the RESEARCH MATERIAL to the receiver solely for the permitted uses and on the terms and conditions set forth in this Agreement. This research material will not be used for commercial purposes such as screening, production or sale for which a commercialization license may be required.

5. RECEIVER agrees that this MATERIAL will not be released to any person other than the signatories of this Agreement except co-workers working directly under a signatory's supervision who have agreed to abide by the terms and conditions of this Agreement. No one is permitted to take or send this RESEARCH MATERIAL to any other location, unless prior written permission is obtained from the PROVIDER; such permission will not be unreasonably withheld.

6. The RECEIVER retains ownership of: (a) modifications which contain/incorporate the RESEARCH MATERIAL (except that, the PROVIDER retains ownership rights to the RESEARCH MATERIAL included therein), and (b) those substances created through the use of the RESEARCH MATERIAL or modifications, but which are not progeny, unmodified derivatives or modifications (those substances that do not contain the original RESEARCH MATERIAL, progeny, unmodified derivatives of the PROVIDER. If either 2 (a) or 2 (b) results from the collaborative efforts of the PROVIDER and the RECEIVER joint ownership may be negotiated.

7. This Agreement and the resulting transfer of RESEARCH MATERIAL constitute a restricted non-exclusive permission for RECEIVER to use the RESEARCH MATERIAL solely for not-for-profit purposes. The RESEARCH MATERIAL will only be used for research purposes as described in the protocol by recipient's investigator in designated laboratory for the research project described in the attached study protocol, under suitable containment conditions. The RESEARCH MATERIAL will not be used in connection with any activity that is subject to consulting or licensing obligations to any third party. Upon completion of the work for which this restricted permission is granted, RESEARCH MATERIAL, which has not been used, will be disposed of as explicitly directed by the PROVIDER. The PROVIDER retains title to the RESEARCH MATERIAL, and RECEIVER shall not obtain any ownership rights in the RESEARCH MATERIAL.

8. RESEARCH MATERIAL is experimental in nature and it is provided AS IS WITHOUT WARRANTY OF ANY SORT, EXPRESSED OR IMPLIED, and INCLUDING WITHOUT LIMITATION WARRANTIES OF MERCHANTABILITY AND FITNESS FOR A PARTICULAR USE. PROVIDER makes no presentation and provides no warrant that the use of the RESEARCH MATERIAL by RECEIVER will not infringe any patent of proprietary rights of third parties.

9. RECEIVER agrees that it will follow all applicable laws and guidelines set forth by proper authority regarding the use and handling of such RESEARCH MATERIAL. The recipient shall notify the provider in writing of any intention, improvement, modification discovery or development to the material or the information made by Recipient or parties, collaborating with Recipient, herein after referred to an "invention". Nothing in this agreement shall, however, be construed as conveying to the provider any rights under any patents or other intellectual property to such invention, and other than as explicitly provided herein. At its option the provider shall be entitled to receive sample of any materials derived from the Materials for its own research and evaluation purposes only.

10. The RECEIVER shall be responsible for any and all import/export requirements and regulations for the reception of such RESEARCH MATERIAL.

11. If the RECEIVER intends to use such RESEARCH MATERIAL to determine if a commercializable system can be developed as a result of the RECEIVER having received this RESEARCH MATERIAL whether patentable or not, RECEIVER shall promptly notify the PROVIDER in writing of the substance of each such discover and of the filing of any patent application thereon. RECEIVER agrees to negotiate in good faith prior to marketing of such discovery compensation to be paid by the RECEIVER to the PROVIDER. Giving consideration to the contributions of the parties to the discovery and its development, such compensation may include royalties in the gross sales values of the worldwide sales of such discovery derived from the MATERIAL.

12. Both RECEIVER and PROVIDER shall provide the other party with a manuscript of any proposed publication or presentation resulting from the study using RESEARCH MATERIAL through the joint project at least sixty (60) days prior to submission thereof for publication or presentation. PROVIDER reserves the right to review any such manuscript and to require the removal of confidential matter in order to protect its proprietary rights and interests. PROVIDER shall notify RECEIVER in writing within the sixty (60) day period concerning the removal of confidential matter and to suggest editorial modifications in the manuscript.

13. Both the PROVIDER and RECEIVER hereby agrees, upon the request, to provide the other party with a report of observations related to the RESEARCH MATERIAL by providing a report describing the results of such research using the RESEARCH MATERIAL. To the extent that it is able, both the PROVIDER and RECEIVER will acknowledge the other party's contribution.

14. The RESERCH MATERIAL is collected under a joint research project and financed by funds received through joint applications of the PROVIDER and the RECEIVER. The PROVIDER maintains, ownership right of the research material and its derivatives unless stated otherwise All information generated using the RESERCH MATERIAL through the joint project is owned by both the PROVIDER and the RECEIVER. Both the PROVIDER and the RECEIVER agrees not to publish results generated through the joint project without citing its source and giving credit of authorship/creatorship to the other counterpart

15. Either party may disclose the other party's Confidential Information to a governmental authority if such party reasonably believes that such disclosure is required by applicable law or regulation or by subpoena or order of court of competent jurisdiction, provided that such disclosure is subject to all applicable governmental or judicial protection available for like material and reasonable advance notice is given to the other party.

16. RECEIVER will exercise all reasonable precautions to protect the integrity and confidentiality of the RESEARCH MATERIAL and RECEIVER shall maintain records

of the location of all MATERIAL. RECEIVER will not remove the RESEARCH MATERIAL from RECEIVER's premises except to the extent necessary to fulfill its obligations under this Agreement

17. This Agreement will terminate: up on completion of current joint research project with the MATERIAL,

18. This Agreement shall be governed by the laws of TANZANIA.

Material Transfer Agreement

Signature page

For Recipient:

Recipient's Investigator	Duly Authorized
____________________	____________________
Signature	Signature/Stamp
_____________________	_____________________
_____________________	_____________________
Date ___________	Date ___________
Mailing Address	Mailing Address for Notices:
_____________________	_____________________
_____________________	_____________________
Tel: _____________________	Tel: _____________________
Fax: _____________________	Fax: _____________________

For Provider:

Provider's Investigator	Duly Authorized
_____________________	_____________________
Signature	Signature
____________________	_____________________
_____________________	_____________________
Date _________________	Date _________________
Mailing Address	Mailing Address for Notices
P.O. Box __________________	P.O. Box _________________
Tel: _____________________	Tel: _____________________
Fax: _____________________	Fax: _____________________
