# Supplementary material for: Efficacy and safety of praziquantel and dihydroartemisinin piperaquine combination for treatment and control of intestinal schistosomiasis: A randomized, non-inferiority clinical trial
Source: PLoS Negl Trop Dis. 2020 Sep 23;14(9):e0008619. doi: 10.1371/journal.pntd.0008619 (PMC7510991; doi:10.1371/journal.pntd.0008619)
Supplement: S1 Diagram — Study participants screening, allocation to treatment and progress in the trial. (DOC) [file pntd.0008619.s003.doc]

**
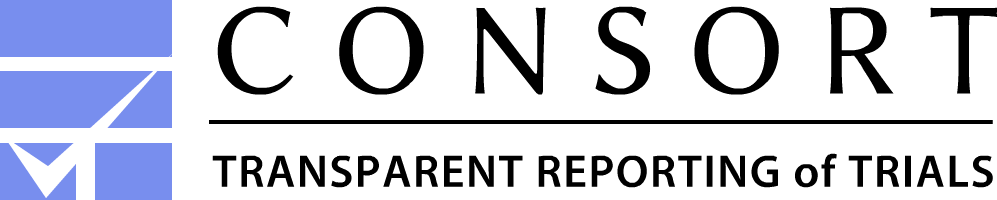
**

**CONSORT Flow Diagram**

**Allocation**

**Analysis**

**Both follow-Up**

**Enrollment**

Total screened (n= 830)

*S. mansoni* negative (n=78)

Assessed for eligibility (752)

Excluded (n= 70)

♦  Not meeting inclusion criteria (n = 0)

♦  Declined to participate before allocation (n = 57)

♦  Other reasons (n = 13)

Analysed (n=341)
♦ Excluded from analysis (n = 0)

Lost to follow-up (n = 0)

Discontinued intervention (n = 0)

PZQ alone (n= 341)

♦ Received allocated intervention (n = 341)

♦ Did not receive allocated intervention (n = 0)

Lost to follow-up (n = 0)

Discontinued intervention (n = 0)

PZQ+DHP (n = 341)

♦ Received allocated intervention (n = 298)

♦ Did not receive allocated intervention due to decline to participate after allocation (n = 48)

Analysed (n= 298)
♦ Excluded from analysis (n = 0)

Randomized (n = 682)
