# Supplementary material for: Efficacy and safety of praziquantel and dihydroartemisinin piperaquine combination for treatment and control of intestinal schistosomiasis: A randomized, non-inferiority clinical trial
Source: PLoS Negl Trop Dis. 2020 Sep 23;14(9):e0008619. doi: 10.1371/journal.pntd.0008619 (PMC7510991; doi:10.1371/journal.pntd.0008619)
Supplement: S1 Table — (DOCX) [file pntd.0008619.s004.docx]

**Table.** Association between baseline characteristics, infection intensity with cure rates within a treatment group at 3 weeks post-treatment visit)

| Variable | Treatment arms | | | | | | | |
| --- | --- | --- | --- | --- | --- | --- | --- | --- |
|  | PZQ arm | | | | PZQ +DHP arm | | | |
|  | **Cured N (%)** | **Not cured N (%)** | ***χ^2^* value** | ***p*-value** | **Cured N (%)** | **Not cured N (%)** | ***χ^2^* value** | ***p*-value** |
| **Age groups** |  |  |  |  |  |  |  |  |
| ≤12 years | 190 (80.9) | 45 (19.1) | 0.72 | 0.79 | 198 (88.0) | 27 (12.0) | 0.058 | 0.81 |
| >12 years | 87 (82.1) | 19 (17.9) |  |  | 65 (89.0) | 8 (11.0) |  |  |
| **Sex** |  |  |  |  |  |  |  |  |
| Male | 128 (80.0) | 32 (20.0) | 0.300 | 0.58 | 130 (89.7) | 15 (10.3) | 0.534 | 0.47 |
| Female | 149 (82.3) | 32 (17.7) |  |  | 133 (86.9) | 20 (13.1) |  |  |
| **Infection intensity** |  |  |  |  |  |  |  |  |
| light | 72 (82.8) | 15 (17.2) | 1.365 | 0.51 | 78 (94.0) | 5 (6.0) | 8.033 | 0.02 |
| Moderate | 126 (82.9) | 26 (17.1) |  |  | 115 (89.8) | 13 (10.2) |  |  |
| Heavy | 79 (77.5) | 23 (22.5) |  |  | 70 (80.5) | 17 (19.5) |  |  |
| **Stunting** |  |  |  |  |  |  |  |  |
| Present | 98 (83.8) | 19 (16.2) | 0.747 | 0.39 | 59 (90.8) | 6 (9.2) | 0.507 | 0.48 |
| Absent | 179 (79.9) | 45 (20.1) |  |  | 204 (87.6) | 29 (12.4) |  |  |
| **Wasting** |  |  |  |  |  |  |  |  |
| Present | 30 (88.2) | 4 (11.8) | 1.215 | 0.27 | 28 (90.3) | 3 (9.7) | 0.143 | 0.71 |
| Absent | 247 (80.5) | 60 (19.5) |  |  | 235 (88.0) | 32 (12.0) |  |  |
