# Supplementary material for: Efficacy and safety of praziquantel and dihydroartemisinin piperaquine combination for treatment and control of intestinal schistosomiasis: A randomized, non-inferiority clinical trial
Source: PLoS Negl Trop Dis. 2020 Sep 23;14(9):e0008619. doi: 10.1371/journal.pntd.0008619 (PMC7510991; doi:10.1371/journal.pntd.0008619)
Supplement: S2 Table — (DOCX) [file pntd.0008619.s005.docx]

**Table**. Association between baseline characteristics, infection intensity with cure rates within treatment group at 8 weeks post-treatment visit)

| Variable | Treatment arms | | | | | | | |
| --- | --- | --- | --- | --- | --- | --- | --- | --- |
|  | PZQ arm | | | | PZQ +DHP arm | | | |
|  | **Cured N (%)** | **Not cured N (%)** | ***χ^2^* value** | ***p*-value** | **Cured N (%)** | **Not cured N (%)** | ***χ^2^* value** | ***p*-value** |
| **Age groups** |  |  |  |  |  |  |  |  |
| ≤12 years | 145 (61.7) | 90 (38.3) | 1.627 | 0.20 | 181 (80.4) | 44 (19.6) | 1.274 | 0.26 |
| >12 years | 73 (68.9) | 33 (31.1) |  |  | 63 (86.3) | 10 (13.7) |  |  |
| **Sex** |  |  |  |  |  |  |  |  |
| Male | 107 (66.9) | 53 (33.1) | 1.134 | 0.29 | 119 (82.1) | 26 (17.9) | 0.007 | 0.93 |
| Female | 111 (61.3) | 70 (38.7) |  |  | 125 (81.7) | 28 (18.3) |  |  |
| **Infection intensity** |  |  |  |  |  |  |  |  |
| light | 55 (63.2) | 32 (36.8) | 1.437 | 0.49 | 73 (88.0) | 10 (12.0) | 4.188 | 0.12 |
| Moderate | 102 (67.1) | 50 (32.9) |  |  | 105 (82.0) | 23 (18.0) |  |  |
| Heavy | 61 (59.8) | 41 (40.2) |  |  | 66 (75.9) | 21 (24.1) |  |  |
| **Stunting** |  |  |  |  |  |  |  |  |
| Present | 78 (66.7) | 39 (33.3) | 0.579 | 0.45 | 56 (86.2) | 9 (13.8) | 1.024 | 0.31 |
| Absent | 140 (62.5) | 84 (37.5) |  |  | 188 (80.7) | 45 (19.3) |  |  |
| **Wasting** |  |  |  |  |  |  |  |  |
| Present | 25 (73.5) | 9 (26.5) | 1.509 | 0.22 | 28 (90.3) | 3 (9.7) | 1.662 | 0.20 |
| Absent | 193 (62.9) | 114 (37.1) |  |  | 216 (80.9) | 51 (19.1) |  |  |
