# Supplementary material for: Efficacy and safety of praziquantel and dihydroartemisinin piperaquine combination for treatment and control of intestinal schistosomiasis: A randomized, non-inferiority clinical trial
Source: PLoS Negl Trop Dis. 2020 Sep 23;14(9):e0008619. doi: 10.1371/journal.pntd.0008619 (PMC7510991; doi:10.1371/journal.pntd.0008619)
Supplement: S3 Table — (DOCX) [file pntd.0008619.s006.docx]

| Eggs count /gram of stool | **Visit** | **PZQ alone** | **PZQ + DHP** |
| --- | --- | --- | --- |
| (Geometric mean ± SD) | At baseline | 194.8±3.5 | 197.3±3.4 |
|  | At 3 weeks visit | 28.1±4.4 | 24.8±3.7 |
|  | At 8 weeks visit | 38.3±3.6 | 31.0±2.5 |

**Table**: Geometric mean intensity at baseline, 3 weeks and 8 weeks post-treatment.
